# Promising Antiviral Activities of Natural Flavonoids against SARS-CoV-2 Targets: Systematic Review

**DOI:** 10.3390/ijms222011069

**Published:** 2021-10-14

**Authors:** Ridhima Kaul, Pradipta Paul, Sanjay Kumar, Dietrich Büsselberg, Vivek Dhar Dwivedi, Ali Chaari

**Affiliations:** 1 Weill Cornell Medicine-Qatar, Education City, Qatar Foundation, Doha 24144, Qatar; rik4001@qatar-med.cornell.edu (R.K.); prp4005@qatar-med.cornell.edu (P.P.); 2 Center for Bioinformatics, Computational and Systems Biology, Pathfinder Research and Training Foundation, Greater Noida 201308, India; sanjay93.sci@gmail.com (S.K.); vivek_bioinformatics@yahoo.com (V.D.D.); 3 School of Biotechnology, Jawaharlal Nehru University, New Delhi 110067, India; 4 Department of Physiology and Biophysics, Weill Cornell Medicine-Qatar, Education City, Qatar Foundation, Doha 24144, Qatar; dib2015@qatar-med.cornell.edu

**Keywords:** flavonoids, coronavirus, SARS-CoV-2, SARS-CoV, MERS-CoV

## Abstract

The ongoing COVID-19 pandemic, caused by the severe acute respiratory syndrome coronavirus 2 (SARS-CoV-2) became a globally leading public health concern over the past two years. Despite the development and administration of multiple vaccines, the mutation of newer strains and challenges to universal immunity has shifted the focus to the lack of efficacious drugs for therapeutic intervention for the disease. As with SARS-CoV, MERS-CoV, and other non-respiratory viruses, flavonoids present themselves as a promising therapeutic intervention given their success in silico, in vitro, in vivo, and more recently, in clinical studies. This review focuses on data from in vitro studies analyzing the effects of flavonoids on various key SARS-CoV-2 targets and presents an analysis of the structure-activity relationships for the same. From 27 primary papers, over 69 flavonoids were investigated for their activities against various SARS-CoV-2 targets, ranging from the promising 3C-like protease (3CLpro) to the less explored nucleocapsid (N) protein; the most promising were quercetin and myricetin derivatives, baicalein, baicalin, EGCG, and tannic acid. We further review promising in silico studies featuring activities of flavonoids against SARS-CoV-2 and list ongoing clinical studies involving the therapeutic potential of flavonoid-rich extracts in combination with synthetic drugs or other polyphenols and suggest prospects for the future of flavonoids against SARS-CoV-2.

## 1. Introduction

In the last two decades, human coronaviruses caused three epidemics: Severe acute respiratory syndrome coronavirus (SARS-CoV-1) in 2003, the Middle East respiratory syndrome coronavirus (MERS-CoV) in 2012, and recently the SARS-CoV-2, responsible for the outbreak of the coronavirus disease 2019 (COVID-19) pandemic, from which the world is suffering since late 2019 [1,2,3]. COVID-19 continuously spreads at a high pace with new strains [4,5]. As of 16 August 2021, there were 207,173,086 confirmed cases of COVID-19, including 4,361,996 deaths, across the globe [4].

**Figure 1:**
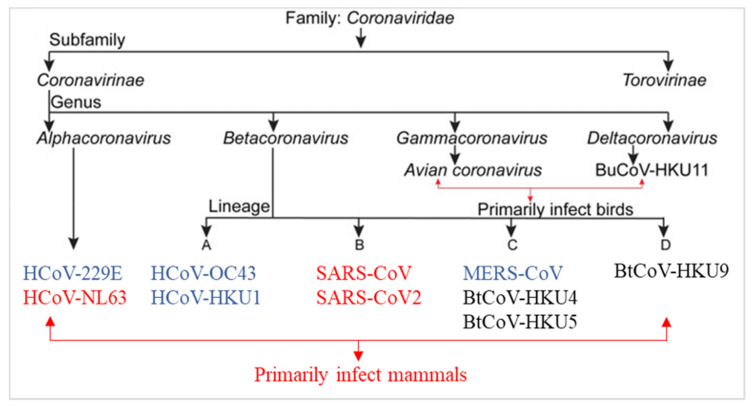
Classification of coronaviruses: the seven known HCoVs are in blue and red. Human coronaviruses (in red) bind the same host receptor, angiotensin-converting enzyme 2 (ACE2).

COVID-19 is associated with severe respiratory symptoms such as pneumonia and is commonly accompanied by fever; it is caused by CoV-2, which belongs to the Beta-coronavirus genus in the family *Coronaviridae* of the order *Nidovirales* [6] (Figure 1). Members of this order share several distinctive characteristics. All of them are surrounded by an envelope that contains very large genomes (≥30 kilobases) characterized by a highly conserved genomic organization. They all express numerous nonstructural genes and replicate using a set of mRNAs [7]. The Beta-coronavirus genus has its ancestor in bat CoVs and includes other viruses such as the human coronaviruses HCoV-OC43 and HCoV-HKU1, which cause the common cold. The genus also includes MERS-CoV and SARS-CoV-1. The latter is closely related to the current CoV-2, though CoV-2 is reported to be more transmissible between individuals [8]. While MERS-CoV and SARS-CoV-1, like SARS-CoV-2, originate from bat CoVs, their intermediate hosts are probably dromedary camels (MERS), civet cats, or raccoon dogs (SARS-CoV-1) [9]. Phylogenetic studies of SARS-CoV-2 genomes and other coronaviruses reveal the phylogenetic relationship among SARS-CoV-2 and other β-CoVs, contributing to the fight with the actual pandemic [10,11,12]. Studies revealed that SARS-CoV-2 (GenBank: MN908947.3) present about 96% nucleotide sequence identity with bat coronavirus RaTG13 (GenBank: MN996532.1), 79.5% identity with SARS-CoV BJ01 (GenBank: AY278488.2) and 55% with MERS-CoV HCoV-EMC (GenBank: MH454272.1) [13]. Based on PubMed, since its emergence, over 100,000 papers have addressed the COVID-19 disease. However, there is still much to learn about SARS-CoV-2 to define efficient therapeutic and/or preventive strategies.

**Figure 2:**
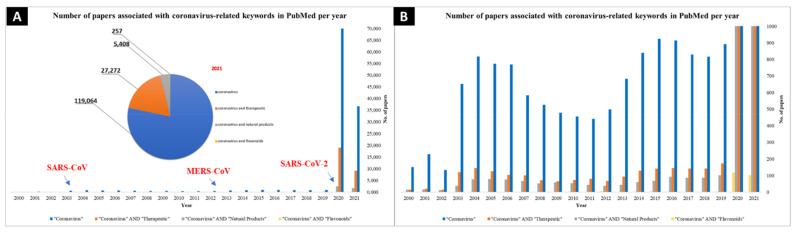
Comparison of the number of papers per year dealing with the words “coronavirus”, “coronavirus and therapeutic”, “coronavirus and natural products” and in the inset, total number of papers used keywords published from 2000 to 2021. (**A**) Bar graph to scale to account for all relevant papers published from 2020–2021, with indications at each major coronavirus outbreak and a pie chart showing the relative number of papers per key phrase. (**B**) Zoomed-in figure to account for low number of hits from 2000–2019 as well the low number of papers dealing with “coronavirus and flavonoids”. (Source: PubMed Database (https://pubmed.ncbi.nlm.nih.gov/), accessed on 3 June 2021).

Despite the progress made in immunization and drug development, global infections keep rising. The highly unequal distribution of vaccines and the development of new COVID-19 strains raised a worldwide effort to find potential inhibitors of key viral processes. Moreover, most antiviral drugs today are single-target drugs designed against a unique viral enzyme. While developing new efficient therapeutic strategies and new drugs is a long process, natural substances are attractive therapeutic solutions in this context. They are proven to be the primary source of antimicrobial and antiviral drugs [14,15]. Many studies have demonstrated that targeting the virus-specific proteins is an effective strategy for drug discovery towards developing direct-acting antivirals [16,17,18]. Based on complementary approaches using both in vitro experiments and in silico virtual screenings, many drug discoveries are initiated by exploiting the effect of natural substances, including flavonoids, on essential coronavirus enzymes as a drug target (Figure 2) [19,20,21,22,23]. While in silico approaches such as molecular dynamics and structure-based virtual screening represent some of the early steps of drug discovery, in vitro studies utilize the findings to narrow down further and experiment with the most promising compounds in cell-based or cell-free methodologies.

Flavonoids represent potential candidates to interfere with the coronavirus life cycle because of their safe administration with lack of systemic toxicity, their ability to work in synergy even with other drugs, and the capacity of their functional groups to interact with different cellular targets and intercept multiple pathways [24,25]. Flavonoids as natural substances represent potential candidates against the actual pandemic because of their biological availability and the participation of most countries (194 countries) on national-level policy for herbal medicines [26]. Moreover, the growing understanding of the efficiency of antiviral drug development based on flavonoids tested previously with other viruses and coronaviruses underlines the importance of exploring these natural products against SARS-CoV-2 [18,24,27]. 

Flavonoids include many secondary metabolites found in fruits, vegetables, and several plants [14,27]. From a chemical view, flavonoids are hydroxylated phenolic molecules characterized by their structural class and degree of hydroxylation. The hydroxyl functional groups of flavonoids are responsible for their antioxidant activity and are formed by two benzene rings (A and B rings), connected via a heterocyclic pyrene ring (C-ring) [28]. Based on their chemical structure, flavonoids are segregated into various classes, among which there are further subclasses [29,30]. They were extensively studied and recently gained increased interest among researchers and clinicians for their antimicrobial, antioxidant, anti-inflammatory, anti-cancer, and antiviral properties with numerous mechanisms to prevent infection and strengthen host immunity [31,32,33,34,35,36,37]. Among their many beneficial effects, antiviral properties can serve as a future therapeutic utility for drugging COVID-19. 

Flavonoids as biologically active substances can affect coronaviruses at the stages of penetration and entry of the viral particle into the cell, replication of the viral nucleic acid, and release of the virion from the cell; they also can act on the host’s cellular targets. These natural compounds could be a vital resource in the combat against coronaviruses, including the actual emerging pandemic. This review highlights the importance of flavonoids and their effects on various key SARS-CoV-2 targets and analyzes the structure–activity relationships for the same.

## 2. Methods

**Figure 3:**
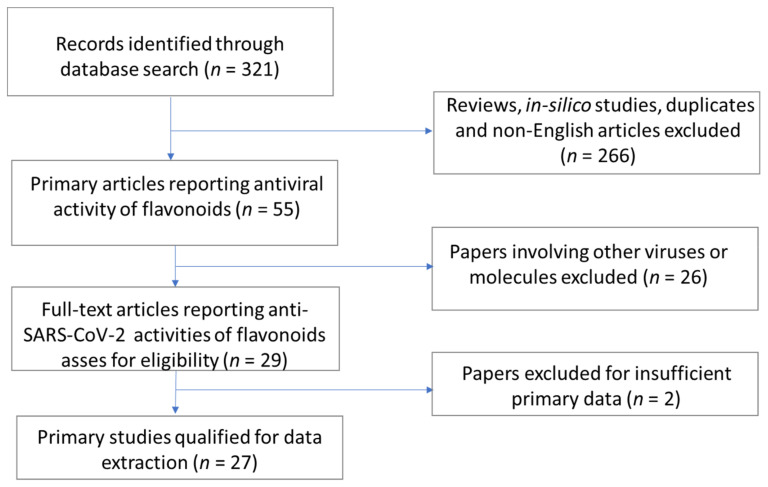
Flow chart of the search strategy.

The review uses references from major databases such as Web of Science, PubMed, Scopus, Elsevier, Springer, and Google Scholar using keywords such as ‘flavonoids’, ‘coronaviruses’, or ‘SARS-CoV’, or ‘MERS-CoV’, or ‘SARS-CoV-2’. An initial search in May 2021 was followed up with another on 17 August 2021 to include any new records that were published. After obtaining all reports from the databases, the papers were analyzed for relevant data; the search protocol is summarized in Figure 3. From 321 search hits, 266 review papers, in silico studies, duplicates, and non-English were removed. From the 55 papers qualified for further screening, 26 articles dealing with other viruses or different compounds were removed from the process.

Moreover, two publications without sufficient primary data on SARS-CoV-2 targets were excluded. Ultimately, 27 primary literature papers exploring the in vitro activities of over 69 flavonoids qualified for the final data extraction phase, with two independent reviewers extracting the data per study. Most studies detailed SARS-CoV-2 3C-like protease (3CLpro) as the most promising target, explaining the large number of flavonoids explored for their respective inhibitory activities against this protease. Other targets included the papain-like protease (PLpro), the spike (S) protein–ACE2 interaction, helicase, and the nucleocapsid (N) protein. Data were segregated according to SARS-CoV-2 target, class of flavonoid, and flavonoid (where multiple studies reported activities for the same flavonoid) and is summarized in tables under relevant sections. Wherever possible, mode of action, efficacy, cytotoxicity, and natural source efficacy of flavonoids are presented.

Nevertheless, other papers reporting general antiviral activities of flavonoids against CoV-2 without target specificity are also discussed. We compiled, evaluated, and analyzed the literature where flavonoids show inhibition against various SARS-CoV-2 targets through the use of various in vitro methodologies. We included both cell-free and cell-based methods, ultimately to suggest follow-up in vivo and clinical trials, focusing on a handful of effective molecules for the treatment of COVID-19. A search using the keywords “COVID-19”, “flavonoids” and “polyphenols” was also conducted on ClinicalTrials.gov to compile and analyze ongoing real-world clinical trials reported until 17 August 2021 that explore the effect of flavonoids on patients suffering from COVID-19.

## 3. Results and Discussion

### 3.1. Coronaviruses Biology and Therapeutic Strategies for the Treatment of COVID-19 Infection

#### 3.1.1. Genomic Characterization and Structure of SARS-CoV-2

**Figure 4:**
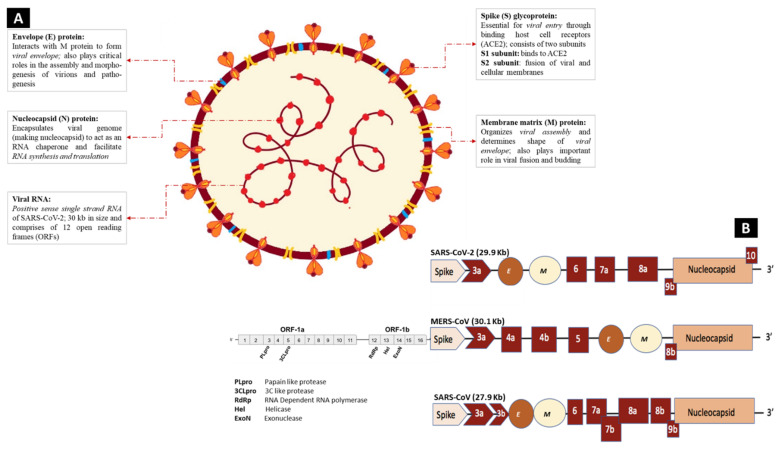
Schematic diagram of the SARS-CoV-2 structural and functional components. (**A**) The single-stranded viral RNA (ssRNA) is encapsulated at places with the nucleocapsid (N) protein, whereas the membrane consists of both the membrane matrix (M) and the envelope (E) proteins, from where the spike (S) protein protrudes outside and interacts with human ACE2 receptors during viral entry. (**B**) Consisting of two open reading frames (ORFs), ORF 1a and 1b, that code for Non-structural proteins (NSPs) as well as PLpro, 3CLpro, RdRp, exonuclease, the 29.9 kb ssRNA genome of SARS-CoV-2 observes similarities with the genomes of previous epidemic-causing coronaviruses, namely MERS-CoV (30.1 kb) and SARS-CoV (27.9 kb). In addition to coding for the polyproteins pp1a and pp1ab, the genome is responsible for translating numerous structural and functional molecules such as the S protein, E protein, M protein, and N protein.

SARS-CoV-2, like other coronaviruses, is enveloped with crown-like particles enclosing a positive-sense single-stranded RNA, which is characterized by a 5′ cap and a 3′ poly (A) tail, which enables it to act as a messenger RNA for translation of replicase polyproteins once inside the host cell [38,39,40]. The single positive strand of SARS-CoV-2 genomic RNA is about 30 kb in size, and like those of SARS-CoV and MERS-CoV, its genome comprises 12 open reading frames (ORFs) in number (Figure 4B) [41].

The first ORF, ORF 1a and 1b, consists of 67% of the genome and encodes RNA polymerase and other nonstructural proteins (nsPs), including the main coronavirus protease chymotrypsin-like protease (3CLpro), RNA-dependent RNA polymerase (RdRp), and papain-like protease (PLpro). At the same time, the remaining ORFs generate several structural and accessory proteins [42,43]. The nonstructural proteins are essential for directing RNA synthesis and processing, cellular mRNA degradation, host immune response suppression, and double-membrane vesicle formation [44,45]. The four main structural proteins encoded by the genomic RNA consist of three main proteins surrounding the viral particle; the surface glycoprotein called the spike protein (S), membrane protein (M), an envelope protein (E), while the fourth nucleocapsid protein (N) is located internally and is intimately associated with the viral genomic RNA (Figure 4A) [41].

The S protein is a trimeric transmembrane glycoprotein responsible for binding with cellular receptors through its S1 subunit and for fusing the virus and the host cell before its entrance with its S2 subunit [46]. Both SARS-CoV and SARS-CoV-2 bind to the same functional host cell receptor, the angiotensin-converting enzyme 2 (ACE2), a membrane protein expressed in the lungs, heart, kidneys, and intestine of the host [13]. The M protein, a 25–30 kDa protein with three transmembrane domains, plays a central role in assembling new viral particles, where host factors and viruses come together to form new virus particles [47]. The E protein, a 9–12 kDa protein, presents an ion channel activity that plays a vital role in the virus life cycle, from assembly to release [47].

N protein, a 45 kDa RNA-binding protein, is the only protein that binds to the viral genome in a beads-on-a-string conformation. This protein is crucial in viral pathogenesis and can cooperate with some of the mentioned structural proteins such as the M protein, which helps to improve the efficiency of virus transcription and assembly [48]. 

#### 3.1.2. Mechanism of Cell Entry and Life Cycle of the Virus

**Figure 5:**
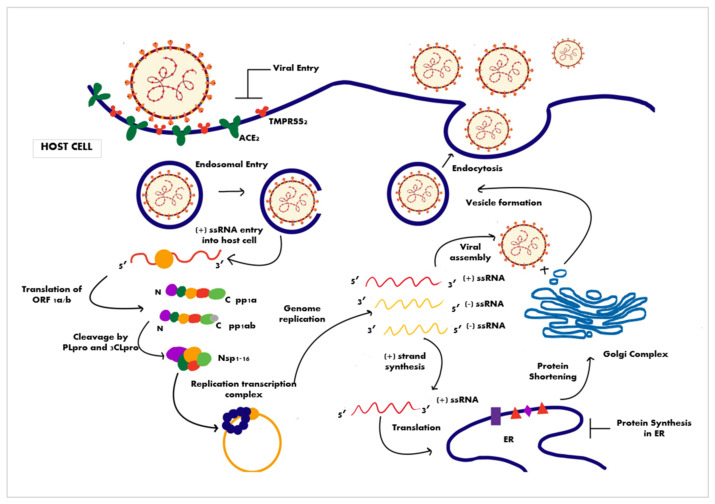
The SARS-CoV-2 viral life cycle with various stages as potential targets of therapeutic intervention.

The virus cell cycle occurs in distinguished steps, including attachment, entry, induction of replicase proteins, replication, transcription, assembly, and discharge of mature viral particles [49] (Figure 5). Receptor binding and membrane fusion are the initial steps facilitated by the attachment of the virus to the host cells via binding of the spike protein (S1 region) to the receptor ACE2 for SARS-CoV and SARS-CoV-2 and the dipeptidyl peptidase 4 receptor (DPP4) for MERS-CoV [49]. This attachment is followed by a series of steps leading to the delivery of the viral genome into the cytoplasm.

Following the binding of the spike to ACE2, host proteases such as transmembrane protease serine 2 (TMPRSS2) are involved in the cleavage of the S protein, which helps the virion enter the host cell. Other proteases, such as Furin, then release the spike fusion peptide, allowing the cellular virus to pass through the endosomal pathway [49]. The virus RNA genome is released into the cytosol of the infected cell. The last step is favored by the low pH of the endosomal microenvironment and the S2 functional subunit of the S spike protein [50]. Once in the cytosol, the genomic RNA is translated into two polyproteins—pp1a and pp1ab—using the host ribosome machinery. The viral genome replication and the synthesis of functional and structural viral proteins occur. These polyproteins undergo proteolytic cleavage by two viral cysteine proteases, PLpro and 3CLpro, generating many nsPs, some of which subsequently assemble to form a replicase–transcriptase complex (RTC) [38,51,52,53]. The RTC, amongst various other domains, contains an RdRp domain which aids the replication of the positive-sense RNA to form a negative-strand RNA intermediate [52,53], which has two different fates. The negative RNA strand can undergo discontinuous transcription to create shorter, usually overlapping, sub-genomic RNA segments. Translation of this sub-genomic RNA produces essential structural proteins such as the M, E, and S proteins, which are then inserted into the secretory pathway of the host cell to be processed and packaged to form virion progeny. Another fate of the negative-strand viral RNA is to undergo further replication to give positive-sense RNA, which is inserted into the virion progeny being processed in the secretory pathway [52,53]. Once processing is completed, the new virion progeny is exocytosed by the cell, and the progeny can infect other host cells [52,53].

#### 3.1.3. Promising Therapeutic Strategies for the Treatment of COVID-19 Infection

**Figure 6:**
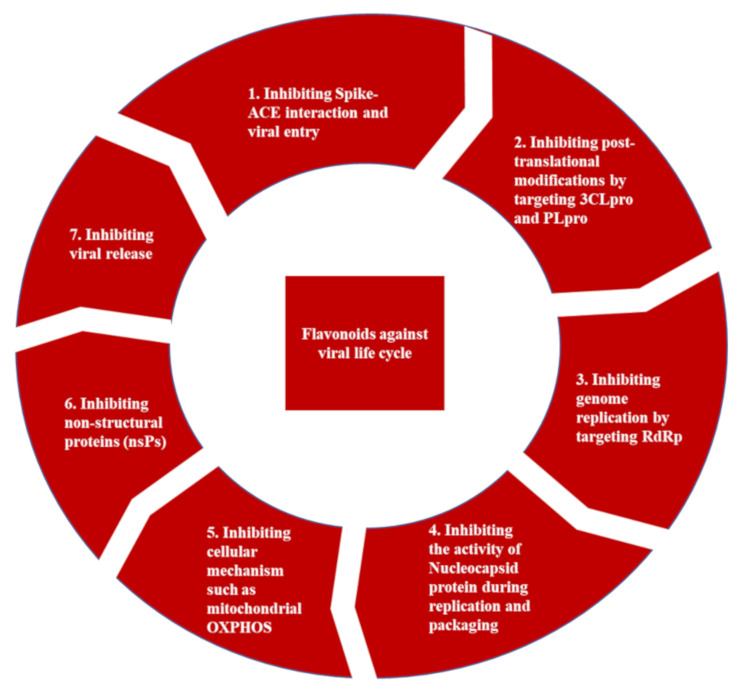
Viral and infected host cell targets important for potential therapeutics for inhibiting the viral pathogenesis of SARS-CoV-2.

Although there is progress against the COVID-19 pandemic following the development of vaccines and some specific drugs that show minor effects against the disease, diverse events have limited this progress. They have raised a worldwide effort to find potential inhibitors relevant to mechanistic targets involved in SARS-CoV-2 infection. Early studies and pharmaceutical experience have shown that antivirals’ de novo development is a time-, cost-, and effort-intensive endeavor [54,55]. Secondly, the safe use of natural compounds derived from natural sources against different viruses, including SARS-CoV and MERS-CoV, was acknowledged for several years, making them potential and powerful anti-COVID-19 drugs [24,51]. In this context, and besides the use and the assays, small molecule drugs, monoclonal antibodies, peptides, and interferon therapies to combat COVID-19, natural products including flavonoids emerged as a safe alternative therapeutic strategy against different targets for blocking the coronavirus life cycle at different stages of viral infection [55]. Flavonoids can directly target specific viral steps and enzymes or components at each phase of the virus life cycle. Figure 6 summarizes different viral and infected host targets that were revealed to be essential to develop potential therapeutics to inhibit the viral pathogenesis of SARS-CoV-2. 

The first therapeutic strategy targets the first step on the virus life cycle, which is the early entry of COVID-19 by interrupting spike–ACE2 protein–protein interaction, TMPRSS2 activity, and endocytic pathway-associated proteins such as clathrin and cathepsin L, preventing the internalization of SARS-CoV-2 in the host cell. The second therapeutic strategy involves the inhibition of the viral proteases 3CLpro and PLpro. The inhibition of these two key proteases blocks the production of non-structural proteins, including the RdRp and helicase, which directly block the transcription and replication of the virus [55]. Moreover, blocking directly viral replication enzymes may consist of the third potential target to treat COVID-19. Finally, the fourth therapeutic strategy targeting the release outside the infected cells of the new virions consists of reducing/silencing the expression and/or the activity of the ion channel viroporin 3a [55].

### 3.2. Flavonoids and Their Antiviral Properties

Flavonoids are the largest group of phenolic phytochemicals in higher plants. They also belong to secondary plant metabolites found in fruits, vegetables, seeds, roots, *propolis*, and other plant products such as tea and wine. With over 9000 structurally identified flavonoids, research has associated many of these compounds with multiple health-promoting effects, ranging from nutraceutical, pharmaceutical, medicinal, and cosmetic applications to their antioxidative, anti-inflammatory anti-mutagenic, and anti-carcinogenic properties [56,57]. In plants, they play an important role as components of cells to defend against pathogens, insects, and other stressful environments [58,59,60,61,62].

**Figure 7:**
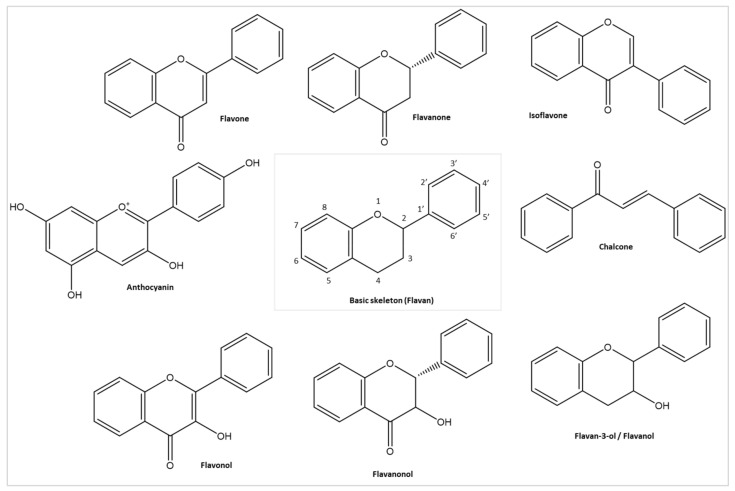
Basic structure of flavonoids (Flavan) and its different classes; image made using ChemDraw (https://perkinelmerinformatics.com/products/research/chemdraw/, accessed on 1 September 2021).

Produced by the phenylpropanoid pathway, flavonoids are hydroxylated phenolic molecules divided into classes by their structure, degree of hydroxylation, and polymerization. As mentioned before, their basic structure (flavan or 2-phenylchroman) consists of two benzene rings (A- and B-rings), connected through a heterocyclic pyrene ring (C-ring). The different subclasses of flavonoids include anthocyanins, chalcones, dihydrochalcones, dihydroflavonols, flavan-3-ols, flavanones, flavones, flavonols, flavanonols, and isoflavonoids (Figure 7).

Unlike synthetic antiviral drugs with narrow antiviral activities and varying levels of patient-specific clinical efficacies, the potent bioactivity and the wide range of pharmacological and toxicological applications of flavonoids, make them be considered as potential therapeutics to both existing and novel public health concerns. In fact, their structures are often inspirations for synthetic therapeutic drugs [63,64,65,66]. Natural extracts and their isolated polyphenols are investigated in depth using in vitro, in vivo, and more recently, in silico studies for their antiviral effects against viral entry to release, as well as the intermediate cascades, such as R/DNA replication, protein translation, and post-translational modifications, of multiple viruses, both respiratory (such as H1N1) and non-respiratory (such as herpes simplex virus [HSV]) [24,49,67,68,69,70,71,72].

#### 3.2.1. Antiviral Activities of Flavonoid against Non-Respiratory Viruses

In 2012, Johari et al. reported the flavone baicalein to have anti-adsorption, anti-replication, and direct virucidal effects against the Japanese encephalitis virus (JEV) **[73]**. Similar experiments by Ting et al. found that flavonol kaempferol and the isoflavone daidzein showed greater anti-JEV properties in pretreated cells [74]. Lani et al. reported that baicalein also repressed the Chikungunya virus (CHIKV) activity, while the glycosylated flavonol quercetagenin inhibited its replication, respectively. Along with the flavonol fisetin, all three inhibit different aspects of viral mechanism: extracellular stage, viral entry, gene replication, and other intracellular stages [75]. Independently, nobiletin and silymarin also were shown to act against CHIKV [76,77]. On the other hand, Zandi et al. showed quercetin to have direct antiviral activity against Dengue virus 2 (DENV-2) [78]. Rutin, baicalein, baicalin, daidzein, and naringenin also exhibited significant inhibition against DENV-2 [79,80,81]. Similarly, against enterovirus A71, the causative agent of encephalitis and hand, foot, and mouth disease (HFMD), apigenin, kaempferol, baicalein, and baicalin showed in vitro and in vivo promise [82,83,84,85]. 

Multiple flavonoids were investigated against the hepatitis C virus (HCV) in the last few decades, given its critical role in a wide array of liver diseases. The flavanone naringenin represses the release of HCV core dose-dependently and reduces its infectivity when pretreated with the flavonoid in vitro [86]. Apigenin, silybin, quercetin, ladanein, sorbifol, and pedalitin are also effective candidates against the same virus [87,88,89,90]. Similar promising concentration-dependent inhibitory effects were reported for epigallocatechin gallate (EGCG) against the Hepatitis B Virus (HBV) e antigen (HBeAg) [89]. Baicalin, genistein, and sodium rutin sulfate (SRS) all have inhibitory properties against the envelope fusion mediated viral entry of human immunodeficiency virus 1 (HIV-1) [89,91,92]. In addition to the above, multiple other naturally occurring and synthetic flavonoids inhibit non-respiratory viruses in the literature [49,72,93,94,95].

#### 3.2.2. Antiviral Activities of Flavonoid against Respiratory Viruses

Imanisi et al. tested epigallocatechin (EGC) as a major constituent of green tea extract (GTE) against Madin-Darby canine kidney (MDCK) cells infected with various strains of the influenzas A and B viruses; the flavanol inhibited the early stages of viral infection in vitro [96]. Similarly, quercetin and its derivatives, silymarin, and multiple other flavonoids also have similar activities [92,97,98,99].

Another common viral pathogen causing respiratory diseases is rhinovirus (RV). Using BEAS-2B cell-based methodologies, quercetin decreases the levels of multiple strains of. Moreover, by pretreating the cells with quercetin before RV infection, viral endocytosis halted significantly, presumably by interaction with and inhibition of the cell enzyme PI-3-kinase; the flavonol potently inhibits the viral replication stage [100]. Additionally, Desideri et al. reported that the novel flavonoid derivatives 6-chloro-3-methoxy-flavone-4′-carboxylic acid, 6-chloro-4′-oxazolinyl, and 6-chloro-3-methoxy-4′-oxazolinyl flavone inhibited the activity of human rhino virus (HRV-1B) without having significant cytotoxic effects on the cells [101]. These examples show great promise in presenting applications of flavonoids against the novel coronavirus (n-CoV).

### 3.3. Antiviral Properties against Coronaviruses, Including SARS-CoV and MERS-CoV

Research exploring the antiviral activities of various natural flavonoids against animal and human coronaviruses span over three decades; some of this primary literature is compiled and reported in Table S1 (see Supplementary Material). In 1990, it was found that kaempferol reduces the replication of both human and bovine coronaviruses in vitro by ~65% at a concentration of just 10 μg/mL; against the same viruses, both chrysin and quercetin inhibited key stages in the replication and infectivity stages, although less effectively [72]. Later, theaflavin constituents (including those with galloyl moieties) of black tea synergistically inhibited bovine coronavirus (BCV) activity [102]. Perhaps most importantly, as priorly reported, quercetin 7-rhamnoside inhibits the non-respiratory coronavirus PEDV with a very potent IC50 = 0.014 μg/mL with high cellular cytotoxic tolerance; its structural analogs also showed promising activities (Table S1) [18]. 

#### 3.3.1. SARS-CoV

SARS-CoV proteases, PLpro and 3CLpro, are the most investigated targets for flavonoid inhibition, some of these are listed in Table S1 [72,103,104,105,106,107,108,109,110,111,112]. In 2010, Ryu et al. demonstrated, using FRET assays, that the biflavonoid amentoflavone, a constituent of *Torreya nucifera*, non-competitively inhibits the SARS-CoV 3CLpro very effectively with an IC50 of 8.3 μM, while three other biflavonoids, namely, bilobetin, ginkgetin, and sciadopitysin, with methylation of 7-, 4′-, and 4′′′-hydroxyl groups, were less potent [103]. The flavonol herbacetin and the flavones rhoifolin and pectolinarin inhibit the protease; their hydrophobic aromatic rings and hydrophilic hydroxyl groups contributing to the binding affinity [104]. Among the seven flavonoids derived from *Pichia pastoris* explored by Nguyen et al., quercetin, ECGC, and GCG were most effective at inhibiting SARS-CoV 3CLpro in vitro; as the best inhibitor, GCG, a flavanol with two galloyl moieties, was further analyzed and was a competitive inhibitor showing a strong affinity for the protease (Table S1) [105]. 

Various polyphenols’ inhibitory effects range from chalcones to flavonols, derived from *Broussonetia papyrifera* on both SARS-CoV cysteine proteases (3CLpro and PLpro). While PLpro inhibition was significantly more potent than 3CLpro overall, the chalcone Broussochalcone A was the most effective in the study [106]. Furthermore, twelve geranylated flavonoids from *Paulownia tomentosa* displayed dose-dependent SARS-CoV PLpro mixed inhibition using fluorogenic assay. While tomentin B, a reversible inhibitor that binds to the active site, was the most potent (lowest Ki; 3.5 μM, mixed), tomentin E had the lowest active concentration marker (IC50 = 5.0 ± 0.06 μM), whereas other constituents of the fruit also had effective concentrations (Table S1) [107].

The flavonol kaempferol’s glycoside derivate, juglanin, inhibited viral production and released through interference with the Ba^2+^-sensitive or cation-selective 3a channel protein of SARS-CoV potently with a very low IC50 of 2.3 µM [108]. Another SARS-CoV target explored is its helicase, nsP13. Scutellarein inhibits the ATPase activity of the helicase very effectively (IC50 = 0.86 ± 0.48 μM), while myricetin, myricitrin, amentoflavone, and Diosmetin-7-O-Glc-Xyl are less potent (Table S1) [109].

Moreover, the anthraquinone emodin, a constituent of the genus *Rheum* and *Polygonum*, inhibited the SARS-CoV S–ACE2 interaction concentration-dependent and also affected the infectivity of SARS-CoV spike-pseudotyped retrovirus to Vero cells [110]. Notably, the glycosylated flavone baicalin has general promising antiviral activity when tested against serum from patients infected with SARS-CoV at different periods post-incubation (Table S1) [111]. Similar experiments revealed that luteolin, procyanidin A2, procyanidin B1, and cinnamtannin B1 have antiviral activities of varying promising efficacies [27,113]. Antiviral activities of flavonoids against the SARS-CoV N protein and NTPase/helicase were also explored and published [114,115].

#### 3.3.2. MERS-CoV

As for SARS-CoV, most flavonoids show anti-MERS-CoV activities targeted its proteases, namely 3CLpro and PLpro. Park et al., using fluorometric cleavage assay, also showed the inhibitory effects of multiple polyphenols derived from Broussonetia papyrifera on MERS-CoV cysteine proteases (3CLpro and PLpro), out of which the chalcone Broussochalcone B showed one of the most effective activities against 3CLpro (IC50 = 27.9 ± 1.2 μM). In contrast, Broussochalcone A was effective against PLpro (IC50 = 42.1 ± 5.0 μM) [106]. Jo et al., using FRET protease assays, reported anti-MERS-CoV 3CLpro activities of herbacetin, isobavachalcone, quercetin 3-β-d-glucoside, and helichrysetin [112]. The literature searches yielded that other flavonoids with lower effectiveness against MERS-CoV viral targets could be potential candidates for better therapeutic interventions against SARS-CoV-2 (Table S1) [116].

### 3.4. Antiviral Activity of Flavonoids against SARS-CoV-2

**Figure 8:**
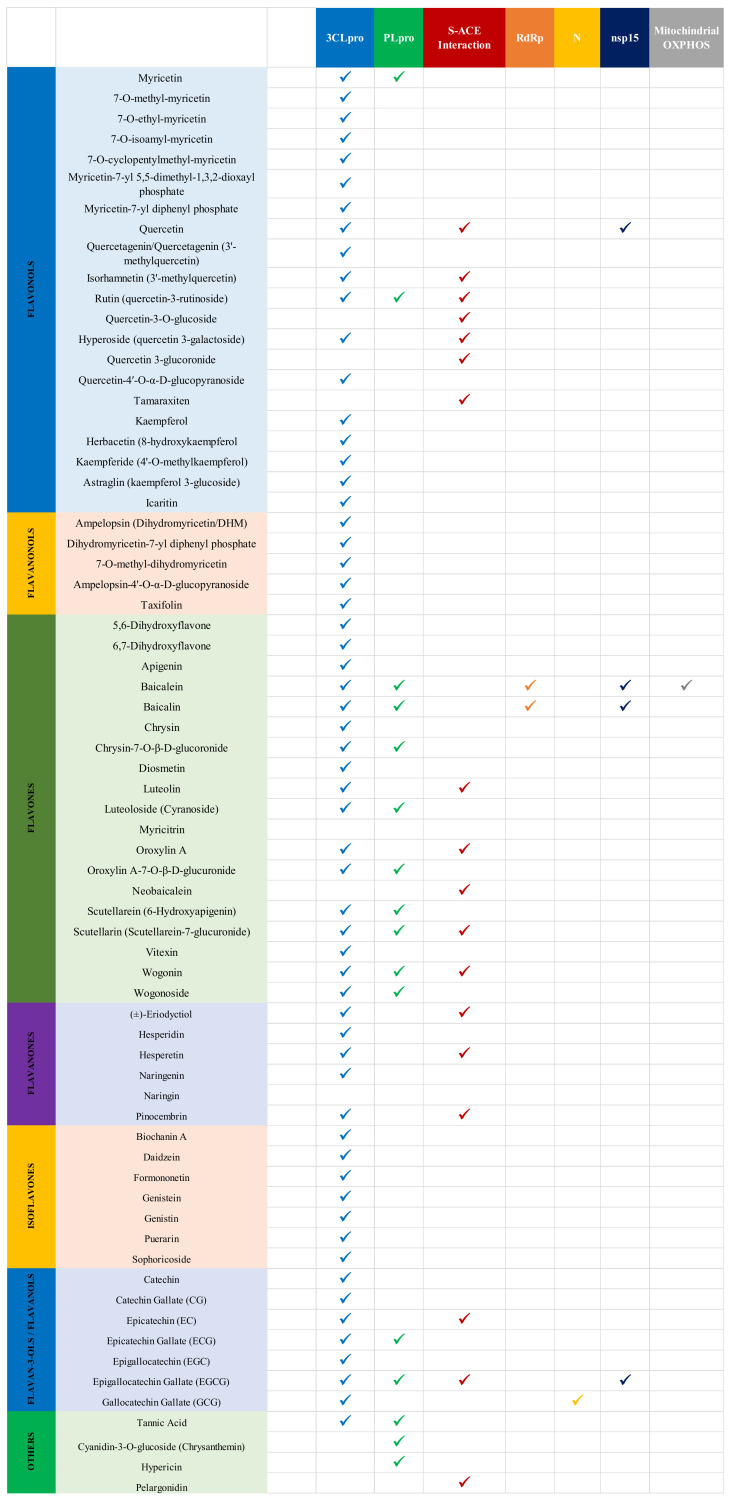
Summary of various flavonoids with anti-SARS-CoV-2 activities, segregated based on subclass and target of inhibition.

More than 69 flavonoids with inhibitory activities against specific SARS-CoV-2 targets were identified, most of whom belonged to the classes of flavonols and flavones, signifying their potent antiviral activities in general (Figure 8). Moreover, the most promising SARS-CoV-2 target was 3CLpro, followed closely by disrupting the S-ACE2 interaction and PLpro. A collection of structures of flavonoids showing anti-SARS-CoV-2 activities are reported in the Supplementary Material (Figures S1–S7), based on subclasses.

#### 3.4.1. Antiviral Activity of Flavonoids against SARS-CoV-2 Proteases (3CLpro and PLpro)

SARS-CoV-2 proteases remain the most popular targets for the antiviral activity of flavonoids. Among these, 3CLpro was investigated against flavonols and flavones more than other subclasses of flavonoids. Table 1 and Table 2 summarize the in vitro activities of various flavonoids against SARS-CoV-2 3CLpro, and PLpro reported in the literature, respectively [117,118,119,120,121,122,123,124,125,126,127,128].

##### Flavonols and Flavanonols

Flavonols have inhibitory activity in vitro against SARS-CoV-2 3CLpro. Of these, myricetin, quercetin, and their derivatives were the largest group of flavonols to have such activity. Among studies reporting the use of cell-based in vitro methodologies, the myricetin C7 derivative, 7-O-methyl-myricetin, was the most effective directly against SARS-CoV-2 3CLpro activity with an IC50 of 0.30 ± 0.00 µM. In contrast, another myricetin derivative, myricetin-7-yl diphenyl phosphate, was the most effective at inhibiting SARS-CoV-2 replication within cells with an EC50 of 3.15 ± 0.84 µM [117]. Among cell-free methods, base myricetin derived from black garlic and *Polygoni avicularis* is effective (IC50 = 43 ± 1 µM) against 3CLpro. Rutin has the lowest IC50 (32 µM); base quercetin also demonstrated a high affinity for the protease active site (Ki = 7.4 µM) [118,124,125].

Multiple flavonols derived from black garlic extract (IC50 = 137 ± 10 µg/mL), among whom quercetin and its derivatives showed remarkable efficacies for 3CLpro, likely due to the flavonols’ interaction with the 3CLpro substrate-binding site as hypothesized and confirmed by independent researchers [117,118,121]. Others reported that quercetin acts as a competitive inhibitor of the 3CLpro active site and has a dose-dependent destabilizing effect on the thermal stability of the protease [124]. In general, the efficacies of most quercetin-derived glycosylated flavonols such as quercetin-4′-O-α-D-glucopyranoside and rutin (quercetin-3-O-rutinoside) against 3CLpro are significantly lower than that of base quercetin at 200 μM when assessed in the same study and under similar conditions [118]. However, other cell-free studies uphold rutin’s status as a potent competitive inhibitor of the 3CLpro catalytic site due to its interaction with the catalytic dyad His41/Cys145 [125]. Similarly, varying cross-study data of quercetagenin, with cell-free and cell-based assays, report drastically different active concentration markers [118,119]. 

In general, the efficacy of myricetin is higher than quercetin, with cell-free methods reporting generally weaker inhibitory capacities against 3CLpro than cell-based assays (Table 1) [117,118,119,122]. Moreover, myricetin is a promising compound given its inhibitory effect against viruses and weak cellular cytotoxicity [117]. Interestingly, many myricetin derivatives, such as ampelopsin (a.k.a. dihydromyricetin or DHM) and its other phosphorylated and glycosylated derivatives, demonstrate inhibition within similar thresholds [117,118]. Other flavonols and flavanonols target SARS-CoV-2 3CLpro less effectively, allowing for vital structure-efficacy relationship analysis (Table 1). 

Lastly, myricetin and rutin possess anti-SARS-CoV-2 PLpro activities, with the latter interacting with the naphthalene inhibitor binding pocket of the protease; however, both of their actions are relatively weak (Table 2) [117,128]. No flavanonols had anti-PLpro activities.

##### Flavones, Flavanones, and Isoflavones

Flavones are another widely explored class of flavonoids for their anti-SARS-CoV-2 activities. *Scutellaria baicalensis* is a traditional Chinese plant that contains several flavonoid elements with antiviral activity. Liu et al. reported that its crude extract, with an IC50 of 8.52 ± 0.54 µg/mL and EC50 of 0.74 ± 0.36 µg/mL against SARS-CoV-2 3CLpro and replication, respectively, has low cellular cytotoxicity and consists of various active flavones [119]. Among these, baicalien was the most effective flavone inhibiting the protease mechanistically by binding to its substrate-binding site and consequently inhibiting viral replication. Two independent groups of researchers using cell-based assays confirm their activities; Liu et al. reported an IC50 value of 0.39 ± 0.12 µM and an EC50 value of 2.92 ± 0.06 µM, while Hai-Xia Su et al. demonstrated an IC50 of 0.94 ± 0.20 µM and an EC50 of 2.49 ± 1.19 µM. Both studies found very high cellular cytotoxic tolerance (>200 µM) to baicalein, showing promise as a therapeutic intervention [119,126]. Its glycosylated derivative, baicalin, and scetullarein are other flavones with promising activities against the SARS-CoV-2 3CLpro, albeit with slightly lower active concentration markers (Table 1).

A proposed mechanism suggests that there are several hydrogen bond interactions between the baicalein and SARS-CoV-2 3CLpro substrate-binding site; these include: the 6-OH of baicalein binds to the carbonyl group of L141, the 7-OH group of baicalein binds to the backbone amide group of G143 and the carbonyl group of baicalein binding to the backbone amide of E166. Additionally, the baicalein molecule covers up the H41 and C145 catalytic residues, adding its inhibitory effect. The higher activity of baicalein compared to baicalin may be attributed to the observation that the larger 7-glycosyl group is more prominent in baicalin, thus reducing effective interaction(s) with the binding site of the protease and consequently lower inhibitory activity. Liu et al. also studied other flavones derived from *Scutellaria baicalensis* extract, including Wogonin (lacks 6-OH and contains 8-methoxy) and Wogonoside (additionally contains C7 glycosylation); however, they showed drastically lower activities, allowing for significant structure-activity relationship analysis [119]. Other flavones extracted from *S. baicalensis* include oroxylin A-7-O-β-D-glucuronide and myricitrin. Overall, the inhibition of PLpro was generally weaker than that of 3CLpro between compounds; no IC50 was reported for this target (Table 2) [126].

Flavanones, as described before, can be characterized as flavones without a C2-C3 double bond on the C-ring. However, their activities against 3CLpro were limited. Nguyen et al. reported naringenin from black garlic extract as the most effective flavanone against SARS-CoV-2 3CLpro with an IC50 of 150 ± 10 µM using cell-free assays. In contrast, Du et al. reported a much higher active concentration for the same compound, likely due to differences in specific targets or methodologies [118,121]. (±)-Eriodictyol, hesperetin, hesperidin (glycosylated hesperetin), and naringin (glycosylated naringenin) are other flavanones that have only moderate to low efficacy against SARS-CoV-2 3CLpro (Table 1) [117,118]. No flavanones exhibited SARS-CoV-2 antiviral activity against PLpro.

Among all the flavonoids extracted from black garlic acid, the most efficacious isoflavone (similar to flavones, but with the B-ring attached to C3 rather than C2) was puerarin with an IC50 value of 42 ± 2 µM, determined using cell-free FRET assays. Daidzein and genistin from black garlic had moderate active concentration markers [116]. Other isoflavones were also studied for their anti-SARS-CoV-2 protease activities (Table 1) [117]. Isoflavones did not have anti-SARS-CoV-2 PLpro activities.

##### Flavan-3-ols/Flavanols

All reports on the activities of flavanols against SARS-CoV-2 3CLpro were performed on cell-free FRET assays. While both Nguyen et al. and Du et al. agree on the status of epigallocatechin gallate (EGCG) as the most promising flavanol against 3CLpro activity, their respective reports on the active concentration marker IC50 vary substantially. The former group reports an IC50 of 171 ± 5 µM for EGCG, whereas the latter group reported an IC50 of 0.847 ± 0.005 µM against the protease while reporting that the flavonoid binds to its substrate-binding site [118,121]. While both cell-free methods, this significant difference might be explained by the difference in material source, specific target, or methodologies. Other catechin derivatives have limited efficacy against 3CLpro; however, an increase in the presence of the galloyl moieties on the flavanol signaled increasing inhibitory activities (Table 1).

Pitsillou et al. reported that flavanols are also moderately effective against SARS-CoV-2 by binding to the naphthalene inhibitor binding pocket and blocking PLpro. The in vitro study used a PLpro enzymatic inhibition assay to investigate the effect of epicatechin gallate (ECG) and epigallocatechin gallate (EGCG) on PLpro. While neither of the compounds showed high activity levels, the study suggests that ECG is slightly more effective at inhibiting PLpro in SARS-CoV-2, revealing essential differences in the structural-activity relationship when comparing anti-3CLpro to anti-PLpro mechanisms (Table 2) [128].

##### Others

Tannic acid, a polyphenol similar to a flavonoid, has the most substantial inhibitory effect on SARS-CoV-2 3CLpro among black garlic constituents with an IC50 of 9 µM using cell-free FRET assay [118]. Like tannic acid, the special polyphenol hypericin has extraordinary potential for its action against the PLpro [128]. Furthermore, other classes of flavonoids, such as anthocyanins contain compounds such as cyanidin-3-O-β-glucoside (Chrysanthemin), which display antiviral activity inhibiting PLpro by binding to the naphthalene inhibitor binding site, although showing limited inhibition (Table 2) [128]. Lastly, Owis et al. upheld the status of a mixture of eleven flavonoids, constituting primarily of the flavonols kaempferol and isorhamnetin derivatives, as having potent cell-based inhibitory activities against 3CLpro using a protease assay and A549 cells. Furthermore, with a lipid encapsulation of the compound, the authors reported significantly higher inhibition (85% vs. 38%), owing to a higher uptake of these otherwise hydrophilic flavonoids through cell membranes (Table 1) [127]. 

#### 3.4.2. Flavonoids against SARS-CoV-2 Spike RBD and hACE2 Interaction

Another promising target for anti-SARS-CoV-2 therapeutic intervention in vitro is the interaction between the viral S protein and the human ACE2 receptor during the viral entry phase. Table 3 summarizes extracted literature data concerning the in vitro activities of various flavonoids against this crucial interaction of the viral life cycle [129,130,131,132].

##### Flavonols

Quercetin and its derivatives are the most predominant flavonols to display in vitro inhibition against the hACE2-spike RBD interaction. Using MCA Fluorescence and rhACE2 cells, Liu et al. reported an effective 66.2 ± 2.2% inhibition at 10 µM and an IC50 of 4.48 µM for base quercetin 2.5 min after incubation with the flavonol against rhACE2 activity, which increased to 29.5 µM after 10.5 min. Independent studies by Zhan et al. confirmed the high affinity of the flavonol through derivation of its Kd (5.92 ± 0.92 μM) and mode of inhibition (mixed) [129,133]. The activities of quercetin derivatives, although relatively limited, have also been explored, allowing for significant structure–activity relationship discussions (Table 3). Among these flavonols, the B-ring 3′,4′-dihydroxylation was responsible for the inhibitory activity; this group is also found in isorhamnetin, explaining similar, although limited, effects [129].

##### Flavones and Flavanones

While few flavones were investigated for disrupting the S-ACE2 interaction, these have shown fewer promising results than previous targets. Luteolin has a 37.1 ± 0.6% inhibition against rhACE2 activity at a low concentration of 10 µM using MCA Fluorescence [129]. Other *Radix Scutellariae*-derived flavones also prevent viral entry into cells through interaction ACE2 and other mechanisms (Table 3) [120]. The flavanones (±)-eriodictyol, hesperetin, and pinocembrin inhibit ACE2–spike interactions, although their effects are weak compared to previously mentioned compounds (Table 3).

##### Flavan-3-ols/Flavanols and Others

EGCG interferes with SARS-CoV-2 spike RBD–ACE2 interaction with the most potent IC50 concentration of IC50 of 2.47 µg/mL, while the flavanol EC, lacking two galloyl moieties, has a much lower inhibitory effect against ACE2 activity [131]. Similar reports are available for EGCG as an effective inhibitor against pseudotyped-SARS-CoV-2 cells with IC50 of 2.47 µg/mL and viral replication and receptor binding [131].

The anthocyanin pelargonidin binds to a fatty acid-binding pocket on spike RBD and attenuates spike–ACE2 interaction, thereby reducing SARS-CoV-2 spike–ACE2 interaction as well as viral replication in Vero cells (Table 3). Pelargonidin also reduces systemic inflammation in in vivo mouse models by interacting with the aryl hydrocarbon receptor (AHR) [132].

#### 3.4.3. Antiviral Activities of Flavonoids against Other SARS-CoV-2 Targets

Table 4 enlists the activities of flavonoids against other less-investigated SARS-CoV-2 targets in various cell-based methodologies.

##### Flavones against SARS-CoV-2 RNA-Dependent RNA Polymerase (RdRp)

The replication stage is an essential step in the viral life cycle, yet few in vitro studies targeted the RdRp to explore the activities of flavonoids. A section of Table 4 summarizes data extracted from the literature concerning the auspicious inhibitory activities of two flavones against this critical enzyme responsible for viral replication. Baicalein and baicalin, flavone constituents of *Scutellaria baicalensis,* were shown to have anti-SARS-CoV-2 RdRp activities. Using Vero and Calu-3 cell-based in vitro methodologies, baicalein more effectively binds to the NiRAN domain and the palm subdomain on SARS-CoV-2 RdRp, thereby inhibiting its activity with an EC50 of 4.5 ± 0.2 μM (Vero cells) and 1.2 ± 0.03 μM (Calu-3 cells) [134]. Cytotoxicity assays showed CC50 values of 86-91 μM, signaling cellular cytotoxic tolerance to the flavone. Similar, although slightly less effective, was its glycosylated analog baicalin (Table 4) [134].

##### Flavan-3-ols/Flavanols against SARS-CoV-2 Nucleocapsid Protein

The nucleocapsid (N) protein is a rare target investigated by researchers to explore the inhibitory effects of flavonoids against SARS-CoV-2 given its location internally within the virus. Table 4 also summarizes data concerning the inhibitory activity of GCG against N protein. This flavanol extracted from green tea was studied in vitro with H1299 cells and qRT-PCR and was effective in blocking N by interfering with N-RNA binding and disrupting its liquid–liquid phase separation (LLPS) with a promising IC50 of 44.4 µM and a CC50 of 156.6 µM [1].

##### Flavonoids against Other SARS-CoV-2 Targets

A green tea extract containing EGCG, EGC, and ECG inhibited SARS-CoV-2 endoribonuclease nsP15 with an IC50 value of 2.54 μg/mL (Table 4). The study isolated the majority constituent of the extract, EGCG, which inhibited SARS-CoV-2 with an IC50 value of 1.62 μM or 0.74 μg/mL (three times lower than that of the extract) by binding to the nsP15 active site [1]. Furthermore, the authors reported anti-nsP15 activities of baicalin, baicalein, and quercetin in vitro with relatively limited, yet promising active concentration markers (Table 4).

Jang et al. studied the effects of EGCG on 3CLpro of human beta coronavirus (HCoV-OC43) and human alpha coronavirus (HCoV-229E) to substitute for SARS-CoV-2. Using a protease assay and qRT-PCR, the authors reported good IC50 values of 14.6 μM against viral production in the beta coronavirus model and 11.7 μM against the alphacoronavirus model. EGCG treatment decreased HCoV-OC43 induced cytotoxicity through plaque formation assays and measurement of changes in cell viability. Moreover, through qRT-PCR, EGCG had inhibitory effects against the beta coronavirus model by reducing the RNA levels of RdRp, membrane protein gene, and nucleocapsid protein gene when cells were conditioned in flavonoid media before and after infection. This points towards EGCG being responsible for inhibiting coronavirus production and transmission, potentially having similar effects on SARS-CoV-2 [137].

On the other hand, a cell-based in vitro study showed that the flavone baicalein inhibits viral replication of SARS-CoV-2 by blocking mitochondrial OXPHOS, which leads to a quick and robust decrease in the mitochondrial membrane potential (MMP) and acts as an oxygen consumption inhibitor [135] (Table 4).

The flavanone Naringenin, at a concentration of 62.5 μM, inhibits HCoV-OC43 by 100% in Vero cells by targeting the endo-lysosomal two-pore channel 2 (TPC2). SARS-CoV-2 infection was inhibited in a time- and dose-dependent manner by naringenin when analyzed for cytopathic effect in Vero cells, while not inducing toxicity on non-infected cells-m signaling towards the selectivity of the flavanone. The authors concluded that naringenin acted as a lysosomotropic active natural compound that exhibited human pan-Coronavirus antiviral activity [138].

Pitsillou et al. used enzymatic assays to explore the inhibition of PLpro deubiquitinase activity in vitro by various small molecules. At 100 μM, the anthraquinone derivative hypericin displayed the highest inhibition of >90%. The anthocyanin cyanidin-3-O-glucoside and the flavanols rutin, EGCG, and ECG followed in terms of their respective PLpro deubiquitinase activities [139].

Using Vero E6 and Calu-3 cells, Leal et al. recently demonstrated that *Siparuna cristata*-derived flavonols, 3,7-Di-*O*-methyl-kaempferol (kumatakenin) and 3,7,3′,4′-Tetra-*O*-methyl-quercetin (retusin), inhibited the general viral replication of SARS-CoV-2 with very promising effective concentrations. Kumatakein showed EC50 values of 10 ± 0.7 and 0.3 ± 0.02 μM in Vero and Calu cells, respectively; similarly, retusin also exhibited promise with EC50s of 0.4 ± 0.05 and 0.6 ± 0.06 μM, respectively [140]. This activity was credited to inhibiting the viral 3CLpro and PLpro due to the authors’ in silico investigations but was not confirmed experimentally.

Song et al. investigated the effects of baicalein from *Scutellaria baicalensis* Georgi on SARS-CoV-2 induced infection parameters. The authors reported that the flavone inhibited cell damage induced by SARS-CoV-2 and improved the morphology of Vero E6 cells at concentrations of 0.1 μM and above. The compound was also studied using hACE2 transgenic mice infected with SARS-CoV-2. Baicalein improves respiratory function and inhibits pulmonary inflammatory cell infiltration while reducing inflammatory cytokines [141].

### 3.5. Structure-Activity Relationships (SARs) of Flavonoids

#### 3.5.1. Effect of Flavonols and Flavanonols on SARS-CoV-2 3CLpro

**Figure 9:**
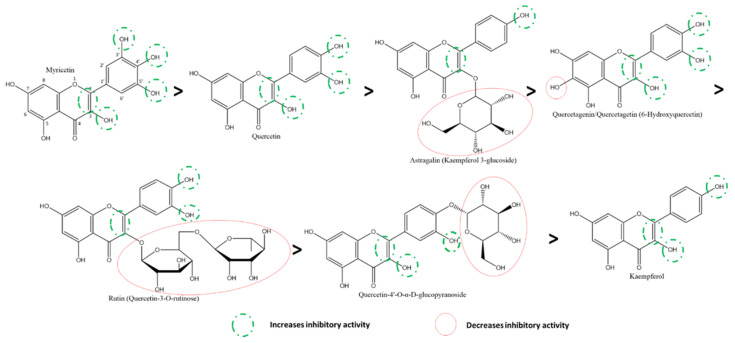
Structure–activity relationships of various flavonols against SARS-CoV-2 3CLpro, adapted from the experiments of [118] using cell-free based in vitro FRET assays. Substitutions and groups that result in an increase/decrease in inhibitory activities are shown in red and green, respectively.

Given the large number of studies exploring the effect of flavonoids on 3CLpro, it is feasible to compare different flavonoids within this class effectively and against this target. Using cell-free assays, Nguyen et al. report that the most to least effective flavonol inhibitors of SARS-CoV-2 3CLpro were myricetin, quercetin, astragalin, quercetagenin, rutin, quercetin-4′-O-α-D-glucopyranoside, kaempferol (Figure 9) [118]. The presence of 3′-OH, 4′-OH, and 5′OH on the B-ring of myricetin accounts for its higher effectiveness compared to quercetin, with an absence of 5′-OH; and kaempferol, with a lack of both 3′-OH and 5′-OH. On the other hand, the presence of A-ring C6-OH accounted for a significant decrease in the inhibitory effect of quercetagenin (6-hydroxyquercetin) compared to quercetin. Further, the addition of carbohydrates to flavonols in place of the hydroxyl groups, as seen in the glycosylation of quercetin (at 4′ on B-ring) to quercetin-4′-O-α-glucopyranoside and (at C3 on C-ring) to rutin (quercetin-3-O-rutinose), decreased the inhibitory effect of these compounds against 3CLpro in vitro. The higher position of astragalin on the spectrum given features such as an absence of B-ring 5′-OH and a presence of C-ring C3 glycosylation leads to the hypothesis that the presence of a hydroxyl on A-ring C6 is detrimental to a flavonol’s activity against 3CLpro despite the presence of both B-ring 4′ and 5′-OH as well as C-ring C3-OH (as seen in quercetagenin). It also suggests that a comparatively larger glycosylated group (such as in rutin) also reduces inhibitory activity significantly.

**Figure 10:**
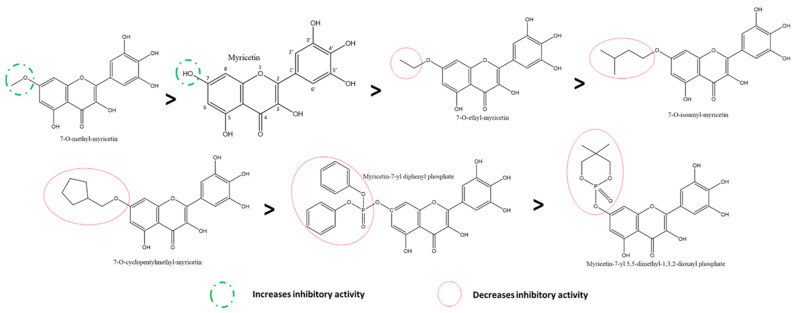
Structure–activity relationships of the flavonols myricetin and its analogs against SARS-CoV-2 3CLpro, adapted from the experiments of [117] using cell-based in vitro FRET assays. Substitutions and groups that result in increase/decrease in inhibitory activities are shown in red and green, respectively.

Comparing the activities of myricetin and its a-ring C7-OH derivates highlights that all of them displayed potent in vitro activities against 3CLpro. The most effective of them was 7-O-methyl myricetin, followed by base myricetin and its ethyl-substituted derivative (Figure 10) [117]. Similarly, by following the trend, isoamyl- and cyclopentylmethyl-derivates show lower inhibition comparatively. In contrast, the largest substitutions, 7-yl diphenyl phosphate and 7-yl 5,5-dimethyl-1,3,2-dioxayl phosphate, were the least effective. This pattern of inhibition in myricetin derivatives shows that, in general, the larger substitutions have lower inhibitory activities against 3CLpro, suggesting a possible hint into the specificity of the binding mechanism.

Further, Owis et al. investigated the inhibitory effects of a mixture of the derivatives of kaempferol and isorhamnetin. The O-glycosylation at C3 of the C-ring in kaempferol glycosylated analogs was the source of the inhibitory activity despite the lower performance of base kaempferol [127].

**Figure 11:**
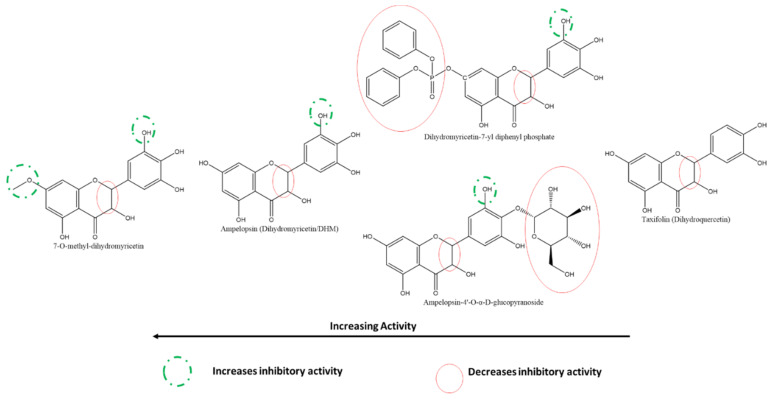
Structure–activity relationships of various flavanonols against SARS-CoV-2 3CLpro, adapted from the in vitro experiments of [117,118]. Substitutions and groups that result in increase/decrease in inhibitory activities are shown in red and green, respectively.

By comparing the inhibition by myricetin to the flavanonol ampelopsin (also known as dihydromyricetin or DHM), it was deduced that the absence of the C-ring C2-C3 double bond further decreased the inhibitory effect, even more as expected, in the B-ring 4′-glycosylated flavanonol, ampelopsin-4′-O-α-D-glucopyranoside (Figure 11) [118]. On the other hand, 7-O-methyl-dihydromyricetin had relatively higher activity than base DHM. In contrast, a larger substitution, such as dihydromyricetin-7-yl diphenyl phosphate, reduced the activity [117]. However, all of these are derivatives of myricetin. Therefore, it is expected to have higher inhibitory activity against Taxifolin (or dihydroquercetin) due to the absence of the B-ring 3′-OH in the latter.

#### 3.5.2. Effect of Flavones and Flavanones on SARS-CoV-2 3CLpro

**Figure 12:**
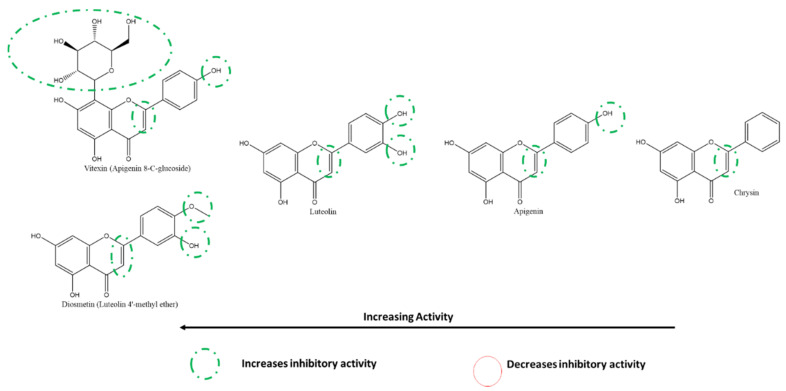
Structure–activity relationships of various flavones against SARS-CoV-2 3CLpro, adapted from the experiments of [118] using cell-free based in vitro FRET assays. Substitutions and groups that result in an increase/decrease in inhibitory activities are shown in red and green, respectively.

Nguyen et al. also reported that at 200 µM, the most to least effective flavone inhibitors of SARS-CoV-2 3CLpro were vitexin > luteolin > apigenin > chrysin (Figure 12). Interestingly, although vitexin does not have a B-ring 5′-OH similar to luteolin, the presence of an A-ring C8-glycosylation resulted in a slightly higher inhibition of 3CLpro. The absence of the glycosylation (in apigenin) along with the absence of a B-ring 5′-OH resulted in a more than 50% decrease in inhibition compared to vitexin. The absence of all hydroxyl groups on the B-ring, including a 4′-OH, (as seen in chrysin) resulted in the lowest inhibition among the flavones in this cell-free based assay [118]. On the other hand, among the flavones investigated by Su et al., diosmetin (the 4′-methoxy derivatives of luteolin) was the only one that showed a slight inhibition at 10 µM. In contrast, apigenin and luteolin failed to show any inhibition, adding to the pattern in flavonols [117].

**Figure 13:**
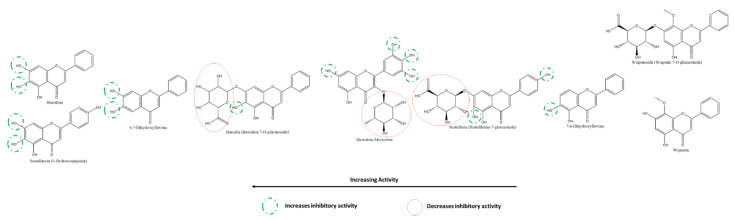
Structure–activity relationships of various flavones against SARS-CoV-2 3CLpro, adapted from the experiments of [119] using cell-based in vitro FRET assays. Substitutions and groups that result in an increase/decrease in inhibitory activities are shown in red and green, respectively.

Liu et al. shed light on the activities of baicalein and its derivates against 3CLpro, and specifically, its substrate-binding site. As described earlier, the A-ring C6 and C7 hydroxyl groups increase their inhibitory effect given that these are responsible for the interaction with 3CLpro. The lower activity of its glycoside derivative, baicalin, is explained by the C7-OH glycosylation, which increases the size of the compound (Figure 13). The activity of scutellarein, slightly lower than baicalein, may be explained by their similarity in structures, although it is interesting to note that scutellarein, with its 4′-OH, has a higher IC50 value. However, its C7 glycoside derivative, scutellarin, has a lower activity than expected, given the reduction in inhibitory activities with glycosylation. The detrimental effects on the inhibitory activity of C-ring C3-glycosylation despite multiple continuous hydroxyl groups on the B-ring are seen in myricitrin. Comparing the actions of 5,6- and 6,7- dihydroxyflavone, one can notice that the presence of hydroxyl on the A-ring C7 position is important for the inhibitory function of flavones. Finally, the derivates wogonin and wogonoside lack the C6-OH, explaining their significantly lower activities [119]. 

**Figure 14:**
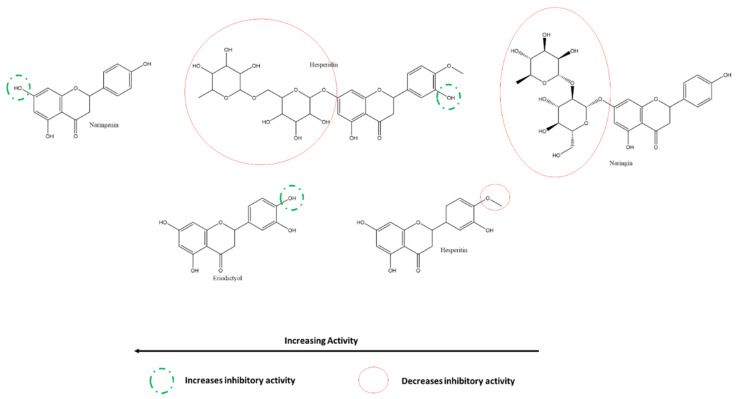
Structure–activity relationships of various flavanones against SARS-CoV-2 3CLpro, adapted from the experiments of [117,118] using in vitro FRET assays. Substitutions and groups that result in an increase/decrease in inhibitory activities are shown in red and green, respectively.

At 200 µM, the most to least effective flavanone inhibitors of SARS-CoV-2 3CLpro are naringenin > hesperidin > naringin (Figure 14) [117]. The A-ring C7-glycosylation of naringenin to naringin translates into more than a three-fold reduction in inhibitory activity. Moreover, being glycosylated at the same atom, hesperidin is much weaker than naringenin; however, it is slightly more inhibitory than naringin. This may be explained by substituting a methoxy group instead of a hydroxyl at the B-ring 4′ position or the mere presence of a hydroxyl group at the B-ring 3′ position in hesperidin rather than in naringin c. On the other hand, the results of Su et al. reveal that methoxy-group substitution at B-ring 4′ position reduces the activity of hesperetin when compared to that of eriodyctiol (Figure 14) [117]. Overall, the differences in inhibition caused by methoxy- group substation at the hydroxyl place are slight and may be attributed to other factors; on the other hand, this variation in patterns provides a better insight into the binding mechanisms of flavanones and other compounds with 3CLpro.

#### 3.5.3. Effect of Flavan-3-ols/Flavanols on SARS-CoV-2 3CLpro

**Figure 15:**
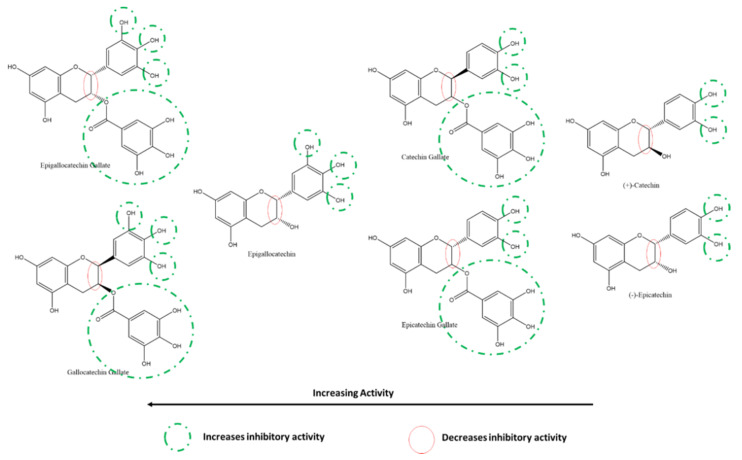
Structure–activity relationships of various flavan-3-ols against SARS-CoV-2 3CLpro, adapted from the experiments of [118]. Substitutions and groups that result in an increase/decrease in inhibitory activities are shown in red and green, respectively.

Among the flavan-3-ols/flavanols, Nguyen et al. reported that at 200 µM, the most to least effective inhibitors of SARS-CoV-2 3CLpro were in the order of EGCG ≈ GCG > EGC > CG = ECG > catechin ≈ EC. Akin to the relationship between myricetin, quercetin, and kaempferol, the presence of 3′, 4′, and 5′ hydroxyl groups on the B-ring of EGCG, GCG and EGC are responsible for their higher SARS-CoV-2 3CLpro inhibitory activity. The galloyl moiety on C-ring C3 of EGCG, GCG, CG, and ECG can also be inferred to increase the inhibitory activity compared to other flavanols such as EGC, catechin, and EC. The effect of 3′-OH is higher than that of the presence of C3-galloyl moiety (Figure 15). However, compared to flavanols, the absence of C-ring C2-C3 double bond and C4 carbonyl on the C-ring of flavanols explains their relative reduced activity [118].

#### 3.5.4. Effect of Isoflavones on SARS-CoV-2 3CLpro

**Figure 16:**
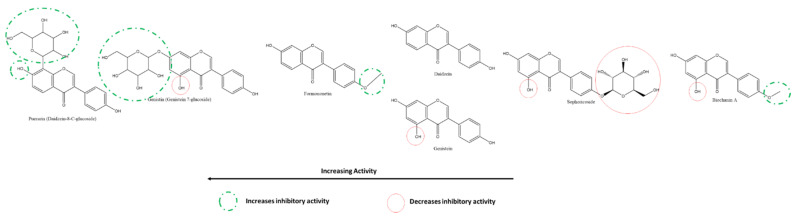
Structure–activity relationships of various isoflavones against SARS-CoV-2 3CLpro, adapted from the experiments of [117,118] using in vitro FRET assays. Substitutions and groups that result in an increase/decrease in inhibitory activities are shown in red and green, respectively.

Nguyen et al. tested the inhibitory activity of three isoflavones, the order of whose activities against SARS-CoV-2 3CLpro were puerarin > daidzein > genistin using cell-free methods in vitro (Figure 16)**.** The presence of A-ring C8-glycosylation in puerarin contributes to its high inhibitory activity. The C8-non-glycosylated daidzein and the C7-glycosylated genistin followed although all three activities were at least two times greater than their flavone analogs [118]. On the other hand, Su et al. also compared a few isoflavones against 3CLpro, revealing that B-ring 4′ glycosylation reduces the inhibitory effect. Substitution of the methoxy-group accounts for the higher inhibition by Formononetin compared to Daidzein and Genistein, whereas the presence of A-ring C5-hydroxyl appears to have the opposite effect [117].

#### 3.5.5. Effect of Flavones on SARS-CoV-2 PLpro

**Figure 17:**
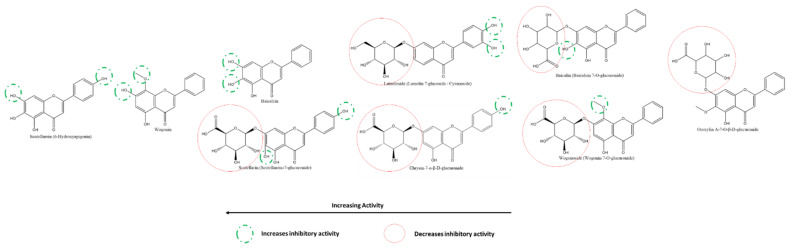
Structure–activity relationships of various flavones against SARS-CoV-2 PLpro, adapted from the experiments of [126] using cell-based in vitro FRET assays. Substitutions and groups that result in an increase/decrease in inhibitory activities are shown in red and green, respectively.

From the cell-based in vitro experiments of Su et al., one can also compare the activities of flavones on SARS-CoV-2 PLpro and consequently analyze the structure–activity relationships of the same (Figure 17) [126]. Scutellarein, with abundant A-ring hydroxyl groups and one on the B-ring, in addition to the absence of any glycosyl-substitutions, showed the highest efficacy against PLpro. As seen from the figure, in general, the presence of glycosylation in place of hydroxyl reduces the inhibitory activities of the flavones; however, this effect is more significant in the presence of hydroxyl groups on the B-ring, which appears to be the source of the anti-oxidative properties of flavonoids, in general. 

#### 3.5.6. Effect of Flavonols on SARS-CoV-2 Spike Protein and hACE2 Receptor Interaction

**Figure 18:**
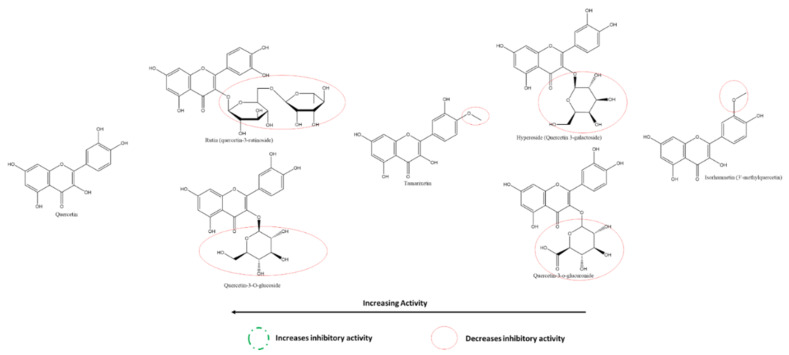
Structure–activity relationships of various flavonols against SARS-CoV-2 S-ACE2 interaction, adapted from the experiments of [129] using cell-based in vitro FRET assays. Substitutions and groups that result in increase/decrease in inhibitory activities are shown in red and green, respectively.

MCA fluorescence and rhACE2 cells determined the inhibitory activities of the flavonols quercetin and its derivates on S protein–ACE2 interaction in vitro. With its multiple hydroxyl groups at the A-, B-, and C-rings, base quercetin was the most potent inhibitor, followed closely by two C-ring C3 glycosides-rutin and isoquercetin, as well as Hyperoside and Quercetin-3-O-glucuronide (Figure 18). Contrary to what was seen for flavonoids against 3CLpro, the substitution of -OH by -OMe at the B-ring of flavonols against S–ACE2 interaction significantly reduces their respective activities [129].

#### 3.5.7. Effect of Flavones on SARS-CoV-2 Spike Protein and hACE2 Receptor Interaction

**Figure 19:**
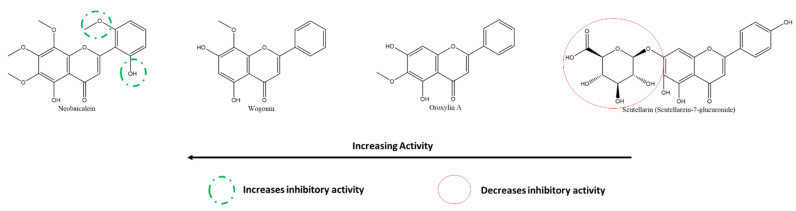
Structure–activity relationships of various flavones against SARS-CoV-2 S-ACE2 interaction, adapted from the experiments of [120] using cell-based in vitro FRET assays. Substitutions and groups that result in an increase/decrease in inhibitory activities are shown in red and green, respectively.

Using HEK293T cells and in vitro assays, Gao et al. showed that the flavones neobaicalein, wogonin, oroxylin A, and scutellarin inhibited the SARS-CoV-2 spike–ACE2 interaction in decreasing order (Figure 19). It can be deduced from their structures that methoxy- and hydroxyl groups on the 1′and 5′ positions at the B-ring, respectively, increased the inhibitory action. In contrast, glycosylation at the A-ring C7 reduced it.

A few general patterns come to light from the analysis of the SARs of the various flavonoids reported in this study. Overall, the presence of hydroxyl groups on all rings, especially on B-ring, of flavonoids increase their respective activities. The most effective flavonoids against 3CLpro, the most promising target, were myricetin, with six hydroxyl groups spread over its three phenolic rings. Secondly, substituting these hydroxyl groups with sugars and other larger groups causes a decrease in inhibitory activity against all respective targets. Moreover, replacing hydroxyl with methoxy-groups resulted in increased activities for many flavonoids, leading to the hypothesis that the polar and electronegative nature of the hydroxyl groups is not the only responsible factor for their high effectiveness. Analysis of the SARs of flavonoids against various targets allows identifying the most effective molecule to test in further stages. Furthermore, it also guides pharmaceutical companies to develop synthetic drugs based on the most inhibiting structural groups in nature.

### 3.6. Flavonoids as Potential Inhibitors of SARS-CoV-2 Proteins: In Silico Studies

In silico approaches in the field of drug discovery such as structure-based virtual screening, molecular dynamics, and absorption, distribution, metabolism, excretion, and toxicity (ADMET) analysis have played an essential role in the screening and identification of various flavonoid inhibitors against the main targets of COVID-19: M^pro^, spike glycoprotein, PL^Pro^, RdRp, helicase and ACE2 within a brief period.

#### 3.6.1. Flavonoids against SARS-CoV-2 M^Pro^

Using molecular docking, Cherrak et al. identified quercetin-3-O-rhamnoside, myricetin 3-rutinoside, and rutin as the potential inhibitors of SARS-CoV-2 M^pro^ in decreasing order with binding energies of −9.7, −9.3, and −9.2 kcal.mol^−1^ respectively [142]. Another report also confirmed that rutin is a potential flavonoid against the SARS-CoV-2 M^Pro^ with a binding energy of −11.187 kcal/mol [143]. A recent study supported the inhibitory effect of rutin on SARS-CoV-2 Mpro via molecular docking with a binding energy −15.63 kcal/mol. Furthermore, ADMET analysis, combinatorial molecular simulations, and hybrid QM/MM approaches concluded that rutin binds very strongly at the active sites of SARS-CoV-2 M^Pro^ by forming three hydrogen bonds at His 163, Glu 166, Gln189 residues [144]. Rakshit et al. screened various flavonoids against M^Pro^ and identified the top five potential flavonoids in order of rhoifolin, 5,7-dimethoxyflavanone-40-O-b-d-glucopyranoside, baicalin, luteolin, and kaempferol based on their binding energies of −9.28, −8.81, −8.29, −8.14, −8.11 kcal/mol [145]. The inhibitory effect of luteolin against the M^pro^ is recently confirmed [146]. Fayyaz et al. screened several flavonoids and identified three potentially active flavonoids, whose activities against SARS-CoV-2 M^pro^ were in the order of rhodiolin > baicalin > silymarin based on their binding energy (−9.05, −8.85, −8.71 kcal/mol respectively) and dissociation constant (0.23, 0.33, 0.41 μm, respectively) using molecular docking and simulation studies [147]. Thioflavonol also inhibits Mpro [148] along with other flavonoids such as apigenin, daidzein, quercetin, kaempferol, luteolin, epigallocatechin, and kaempferol using molecular docking and simulation analysis [104].

The M^Pro^ is considered the most promising drug target for SARS-CoV-2 due to its proteolytic activity, cleaving viral polyprotein into independent functional proteins required for SARS-CoV-2 replication [149,150,151]. The other reason for its therapeutic importance is its dissimilarity to any human cell protease [152,153,154] and its similarity with the M^Pro^ of SARS-CoV [155]. The most common active site residues of M^Pro^ were Glu166, His163, and Met165, which were involved in the interaction with most of the flavonoids. 

#### 3.6.2. Flavonoids against SARS-CoV-2 Spike Glycoprotein

Rutin inhibits the SARS-CoV-2 spike glycoprotein but with less binding energy (−7.9 kcal/mol) [143]. On the other hand, naringin inhibits the spike glycoprotein more effectively with binding energy −9.8 kcal/mol compared to standard drug dexamethasone with the binding energy of −7.9 kcal/mol [156]. Fayyaz et al. described potentially active flavonoids, whose activities against SARS-CoV-2 spike protein were in the order of rhodiolin > hesperidin (with active site 1) > hesperidin (with active site 2) > silyhermin based on their binding energy (−8.68, −8.53, −8.18, −8.05 kcal/mol, respectively) and dissociation constant (0.43, 0.56, 1.01, 1.25 μm respectively) [147]. The authors showed that hesperidin could bind to two different active sites on the spike glycoprotein with different binding energies. Teli et al. highlighted that rutin could serve as a dual receptor inhibitor against the Mpro and spike glycoprotein of SARS-CoV-2 with improved ADMET parameters.

#### 3.6.3. Flavonoids against SARS-CoV-2 PL^Pro^

Potentially active flavonoids, whose activities against SARS-CoV-2 PL^Pro^ were in the order of baicalin > hesperidin > naringen > flemiflavanone D > Euchresta flavanone A on the basis of their binding energy (−10.82, −10.61, −10.17, −10.07, −9.95 kcal/mol, respectively) and dissociation constant (0.01, 0.02, 0.04, 0.04, 0.05 μm, respectively) using molecular docking and simulation studies [147]. The active site residues of PL^Pro^, which are found common with almost all the flavonoids were Lys157, Leu162, Gly163, Asp164, and Glu167, and therefore, all these residues are critical for the ligand interaction.

#### 3.6.4. Flavonoids against SARS-CoV-2 RdRp

An interesting protein–ligand blind docking approach proposed that the SARS-CoV-2 RNA replication can be inhibited by targeting its RdRp protein. Theaflavin inhibits the viral RNA replication by interfering with the RdRp catalytic pocket with the binding energy of −8.8 kcal/mol [157]. Shawan et al. identified luteolin as a potential inhibitor of ACE2 with the binding energy of −10.1 kcal/mol, which is very close to the binding energy of FDA-approved remdesivir (−10.0 kcal/mol) [158]. Fayyaz et al. identified three potentially active flavonoids against SARS-CoV-2 RdRp were in the order of hesperidin > baicalin > naringen based on their binding energy (−9.53, −9.01, −8.54 kcal/mol, respectively) and dissociation constant (0.1, 0.25, 0.55 μm, respectively) using molecular docking and simulation studies [147].

#### 3.6.5. Flavonoids against SARS-CoV-2 Helicase

Along with Mpro, PL^Pro^, RdRp, and spike protein, Fayyaz et al. identified two potential flavonoids: hesperidin and baicalin based on binding energies (−8.93 and −8.9 kcal/mol, respectively) and dissociation constant (0.283 and 0.29 μM, respectively). Hesperidin and baicalin are the only flavonoids that interact and inhibit all the main targets of SARS-CoV-2, such as M^Pro^, PL^Pro^, RdRp, and helicase with excellent binding energies; therefore, both of these flavonoids are considered as multi receptor/protein targets for COVID-19.

#### 3.6.6. Flavonoids against SARS-CoV-2 ACE2

Luteolin is a potential inhibitor of ACE2 with the binding energy of −10.1 kcal/mol, which is very close to the binding energy of FDA-approved remdesivir (−10.0 kcal/mol) [158]. Using virtual screening via Autodock vina, studies identified various flavonoids such as Myritilin, myricitrin, δ-Viniferin, TaiwanhomoflavoneA, Afzelin Biorobin, and Nympholide A that can inhibit the ACE2 [159]. Similarly, Hesperetin, Baicalin, Scutellarin against ACE2 using virtual screening and molecular docking studies [160]. Tangeretin, Nobiletin, Naringenin, Brazilein, Brazilin, Galangin also inhibit ACE2 receptors [161].

### 3.7. Clinical Trials and Future Prospects

Many in vitro studies exploring the anti-SARS-CoV-2 action of flavonoids were published over the last two years since the advent of the COVID-19 pandemic. These studies were guided by in silico studies and the further in vitro or in vivo research of anti-SARS-CoV and anti-MERS-CoV activities of various flavonoids over the last decade owing to the two epidemics caused by these respective. 

Until 17 August 2021, 13 clinical trials were reported and explored the effect and efficacy of various flavonoids and a few other polyphenols and their extracts on COVID-19 patients (Table 5 and Table S2). Of these, the most promising and popular flavonoid is the flavonol quercetin, furthering our findings. In particular, the study (NCT04401202) exploring the effect of *Nigella sativa* seed oil, which is rich in quercetin and kaempferol [162], found in its phase 2 trial that 62.1% of the intervention group which received *Nigella sativa* oil 500mg soft gel capsules orally twice daily recovered within 14 days, compared to only 36% in the control group. Following their progress can prove highly beneficial to clinicians around the globe in identifying potential COVID-19 therapeutic agents at the earliest.

## 4. Conclusions

The results of our review of in vitro and in silico studies are encouraging. Considering that no internationally accepted effective therapeutic intervention exists for COVID-19, there is an urgent need for more extended studies and further in vivo and clinical trials to confirm these results and promote the synthesis of more efficient drugs using the SARs outlined in previous sections. 

In this review, we report that the flavonols quercetin, myricetin, and their derivatives, the flavones baicalin and baicalein, the flavan-3-ol EGCG, and finally, tannic acid, have the most promising scope for further evaluation using both in vivo and consequently clinical studies. Unfortunately, the tendency of flavonoids to aggregate and their limited bioavailability limit their therapeutic interventions. The use of flavonoids in combination with synthetic and commercially produced drugs showed promising results, but more research is needed to prove their synergistic effects. Looking at the growing concern of antiviral resistance, naturally occurring flavonoids are a promising alternative.

## Figures and Tables

**Table 1:** Flavonoids with antiviral activities reported against SARS-CoV-2 3CLpro using in vitro methodologies segregated according to class.

Class	Class Flavonoid	Natural Source (N.S.)/Extract	Efficacy of N.S. Extract	Mode of Action	Methods Used	IC50 (µM)	EC50 (µM)	% Inhibition	Reference
Flavonol	7-O-methyl-myricetin			Interacts with 3CLpro catalytic site	FRET assay, Vero E6 Cells, qRT-PCR	0.30 ± 0.00	12.59 ± 4.41		[117]
	7-O-ethyl-myricetin			Interacts with 3CLpro catalytic site	FRET assay, Vero E6 Cells, qRT-PCR	0.74 ± 0.06	51.01 ± 12.79		[117]
	7-O-isoamyl-myricetin			Interacts with 3CLpro catalytic site	FRET assay, Vero E6 Cells, qRT-PCR	1.92 ± 0.16	31.54 ± 0.74		[117]
	7-O-cyclopentylmethyl-myricetin			Interacts with 3CLpro catalytic site	FRET assay, Vero E6 Cells, qRT-PCR	2.45 ± 0.26	7.56 ± 2.34		[117]
	Astragalin (kaempferol 3-glucoside)	Black garlic extract	IC50: 137 ± 10 µg/mL, 100% inhibition at 0.5 mg/mL	Inhibits 3CLpro activity	FRET assay	143 ± 9		61 at 200 µM	[118]
	Herbacetin (8-hydroxykaempferol)	Flaxseed hulls, *Rhodiola*		Binds to the 3CLpro substrate binding site	Vero cells/RT PCR			59.1 ± 1.9 at 50 µM	[119]
	Hyperoside (quercetin 3-galactoside)	*Nelumbo nucifera*		Interacts with 3CLpro catalytic site	FRET assay, Vero E6 Cells, qRT-PCR			5.2 at 10 µM	[117]
	Icaritin	Black garlic extract	IC50: 137 ± 10 µg/mL 100% inhibition at 0.5 mg/mL	Inhibits 3CLpro activity	FRET assay			31 at 200 µM	[118]
	Isorhamnetin (3-methylquercetin)	Pears, olive oil, wine		Interacts with 3CLpro catalytic site	FRET assay, Vero E6 Cells, qRT-PCR			−2.6 at 10 µM	[117]
	Kaempferol	Black garlic extract	IC50: 137 ± 10 µg/mL 100% inhibition at 0.5 mg/mL	Inhibits 3CLpro activity	FRET assay			16 at 200 µM	[118]
		TCM		Binds to 3CLpro active site	Vero E6 Cells	34.46			[120]
	Kaempferide			Interacts with 3CLpro catalytic site	FRET assay, Vero E6 Cells, qRT-PCR			8.1 at 10 µM	[117]
		TCM		Binds to the 3CLpro substrate binding site.	FRET assay	>100			[121]
	Myricetin	Black garlic extract	IC50: 137 ± 10 µg/mL 100% inhibition at 0.5 mg/mL	Inhibits 3CLpro activity	FRET assay	43 ± 1		80 at 200 µM	[118]
		*Polygoni* *avicularis*		Binds to the 3CLpro substrate binding site	Vero cells/RT PCR	2.86 ± 0.23			[119]
				Positions itself in the 3CLpro binding pocket	FRET assay, BEAS-2B cells	3.684 ± 0.076		97.79 at 50 µM	[122]
				Binds at the catalytic site within the extended substrate-binding pocket	FRET assay, Vero E6 Cells, qRT-PCR	0.63 ± 0.01	8.00 ± 2.05	97.6 at 10 µM	[117]
		*Ampelopsis grossedentata* extract	99.74% inhibition at 100 μg/mL IC50 = 3.44 μg/mL	Modify key residue in domain III of 3CLpro	FRET assay	1.21 (60 min pre-incubation) 21.44 (0.5 min pre-incubation)			[123]
	Myricetin-7-yl 5,5-dimethyl-1,3,2-dioxayl phosphate			Interacts with 3CLpro catalytic site	FRET assay, Vero E6 Cells, qRT-PCR	6.62 ± 0.42	33.45 ± 11.96		[117]
	Myricetin-7-yl diphenyl phosphate			Interacts with 3CLpro catalytic site	FRET assay, Vero E6 Cells, qRT-PCR	3.13 ± 0.37	3.15 ± 0.84		[117]
	Quercetagenin/quercetagenin	Black garlic extract	IC50: 137 ± 10 µg/mL 100% inhibition at 0.5 mg/mL	Inhibits 3CLpro activity	FRET assay	145 ± 6		58 at 200 µM	[118]
		*Eriocaulon* *buergerianum*		Binds to the 3CLpro substrate binding site	Vero cells/RT PCR	1.24 ± 0.14			[119]
	Quercetin	Black garlic extract	IC50: 137 ± 10 µg/mL 100% inhibition at 0.5 mg/mL	Inhibits 3CLpro activity	FRET assay	93 ± 5		74 at 200 µM	[118]
		TCM		Binds to the 3CLpro substrate binding site.	FRET assay	97.460 ± 2.263			[121]
				Interacts with 3CLpro catalytic site	FRET assay, Vero E6 Cells, qRT-PCR			41.3 at 10 µM	[117]
				Binds to SARS-CoV-2 3CLpro active site	FRET assay	Kiapp = 21 μM Ki = 7.4 μM Kd = 2.7 μM (no NaCl) Kd = 150 mM (150 mM NaCl)			[124]
	Quercetin-4′-O-α-D-glucopyranoside	Black garlic extract	IC50: 137 ± 10 µg/mL 100% inhibition at 0.5 mg/mL	Inhibits 3CLpro activity	FRET assay			26 at 200 µM	[118]
	Rutin (quercetin-3-O-rutinose)	Black garlic extract	IC50: 137 ± 10 µg/mL 100% inhibition at 0.5 mg/mL	Inhibits 3CLpro activity	FRET assay			45 at 200 µM	[118]
				Binds to the 3CLpro catalytic site.	FRET assay	32		43 at 30 µM 65 at 60 µM 80 at 120 µM	[125]
Flavanonol	Ampelopsin (dihydromyricetin/DHM)	Black garlic extract	IC50: 137 ± 10 µg/mL 100% inhibition at 0.5 mg/mL	Inhibits 3CLpro activity	FRET assay	128 ± 5		64 at 200 µM	[118]
		*Ampelopsis japonica*		Binds to the 3CLpro substrate binding site	Vero cells/RT PCR	1.20 ± 0.09			[119]
				Interacts with 3CLpro catalytic site	FRET assay, Vero E6 Cells, qRT-PCR	1.14 ± 0.03	13.56 ± 2.50	93.8 at 10 µM	[117]
		*Ampelopsis grossedentata* extract	99.74% inhibition at 100 μg/mL IC50 = 3.44 μg/mL	Modify key residue in domain III of 3CLpro	FRET assay	4.91 (60 min pre-incubation) 34.61 (0.5 min pre-incubation)			[123]
	Isodihydromyricetin	*Ampelopsis grossedentata* extract	99.74% inhibition at 100 μg/mL IC50 = 3.44 μg/mL	Modify key residue in domain III of 3CLpro	FRET assay	3.73 (60 min pre-incubation) 29.04 (0.5 min pre-incubation)			[123]
	Dihydromyricetin-7-yl diphenyl phosphate			Interacts with 3CLpro catalytic site	FRET assay, Vero E6 Cells, qRT-PCR	1.84 ± 0.22	9.03 ± 1.36		[117]
	7-O-methyl-dihydromyricetin			Interacts with 3CLpro catalytic site	FRET assay, Vero E6 Cells, qRT-PCR	0.26 ± 0.02	11.50 ± 4.57		[117]
	Ampelopsin-4′-O-α-D-glucopyranoside	Black garlic extract	IC50: 137 ± 10 µg/mL 100% inhibition at 0.5 mg/mL	Inhibits 3CLpro activity	FRET assay	195 ± 5		50 at 200 µM	[118]
	Taxifolin			Interacts with 3CLpro catalytic site	FRET assay, Vero E6 Cells, qRT-PCR			28.0 at 10 µM	[117]
		*Ampelopsis grossedentata* extract	99.74% inhibition at 100 μg/mL IC50 = 3.44 μg/mL	Inhibits 3CLpro activity	FRET assay	72.72 (60 min pre-incubation)			[123]
Flavanone	(±)-Eriodyctiol			Interacts with 3CLpro catalytic site	FRET assay, Vero E6 Cells, qRT-PCR			34.5 at 10 µM	[117]
	Hesperidin	Black garlic extract	IC50: 137 ± 10 µg/mL 100% inhibition at 0.5 mg/mL	Inhibits 3CLpro activity	FRET assay			22 at 200 µM	[118]
	Hesperetin			Interacts with 3CLpro catalytic site	FRET assay			13.8 at 10 µM	[117]
	Naringenin	Black garlic extract	IC50: 137 ± 10 µg/mL 100% inhibition at 0.5 mg/mL	Inhibits 3CLpro activity	FRET assay	150 ± 10		57 at 200 µM	[118]
		TCM		Binds to the 3CLpro substrate binding site.	FRET assay	>1000			[121]
	Naringin	Black garlic extract	IC50: 137 ± 10 µg/mL 100% inhibition at 0.5 mg/mL	Inhibits 3CLpro activity	FRET assay			18 at 200 µM	[118]
Flavones	5,6-dihydroxyflavone			Binds to the 3CLpro substrate binding site	Vero cells/RT PCR			26.6 ± 0.4 at 50 µM	[119]
	6,7-dihydroxyflavone			Binds to the 3CLpro substrate binding site	Vero cells/RT PCR			56.7 ± 2.0 at 50 µM	[119]
	Apigenin	Black garlic extract	IC50: 137 ± 10 µg/mL 100% inhibition at 0.5 mg/mL	Inhibits 3CLpro activity	FRET assay			25 at 200 µM	[118]
				Interacts with 3CLpro catalytic site	FRET assay			−1.0 at 10 µM	[117]
	Baicalein	*Scutellaria* *baicalensis*	IC50: 8.52 ± 0.54 µg/mL EC50: 0.74 ± 0.36 µg/mL CC50: > 500 µg/mL	Binds to the 3CLpro substrate binding site	Vero cells/RT PCR	0.39 ± 0.12	2.92 ± 0.06		[119]
				Binds to the core of the substrate-binding pocket, preventing substrate access to the active site	Vero E6 cells/CCK8 assays/qRT-PCR	0.94 ± 0.20	2.49 ± 1.19	99.4 at 100 µM 87 at 10 µM	[126]
	Baicalin	*Scutellaria* *baicalensis*	IC50: 8.52 ± 0.54 µg/mL EC50: 0.74 ± 0.36 µg/mL CC50: > 500 µg/mL	Binds to the 3CLpro substrate binding site	Vero cells/RT PCR	83.4 ± 0.9		41.5 ± 0.6 at 50 µM	[119]
				Binds to 3CLpro active site	Vero E6 cells/CCK8 assays/qRT-PCR	6.41 ± 0.95	27.87 ± 12.5	97.6 at 100 µM 68.9 at 10 µM	[126]
	Chrysin	Black garlic extract	IC50: 137 ± 10 µg/mL 100% inhibition at 0.5 mg/mL	Inhibits 3CLpro activity	FRET assay			9 at 200 µM	[118]
				Binds to the 3CLpro substrate binding site.	Vero cells/RT PCR			2.6 ± 1.1 at 50 µM	[119]
	Chrysin-7-O-β-D-glucoronide	*Scutellaria* *baicalensis*		Binds to 3CLpro active site	Vero E6 cells/CCK8 assays			50.6 at 100 μM 24.2 at 10 μM	[126]
	Diosmetin			Interacts with 3CLpro catalytic site	FRET assay			11.3 at 10 µM	[117]
	Luteolin	Black garlic extract	IC50: 137 ± 10 µg/mL 100% inhibition at 0.5 mg/mL	Inhibits 3CLpro activity	FRET assay			45 at 200 µM	[118]
		TCM		Binds to the 3CLpro substrate binding site.	FRET assay	89.670 ± 4.712			[121]
				Interacts with 3CLpro catalytic site	FRET assay			−4.1 at 10 µM	[117]
	Luteoloside (cyranoside)	*L. japonica*		Binds to 3CLpro active site	Vero E6 cells/CCK8 assays			65.4 at 100 μM 14.8 at 10 μM	[126]
	Myricitrin	*Polygoni avicularis*		Binds to the 3CLpro substrate binding site	Vero cells/RT PCR			30.8 ± 4.6 at 50 µM	[119]
		*Ampelopsis grossedentata* extract	99.74% inhibition at 100 μg/mL IC50 = 3.44 μg/mL	Modify key residue in domain III of 3CLpro	FRET assay	14.22 (60 min pre-incubation)			[123]
	Oroxylin A-7-O-β-D-glucuronide	*Scutellaria* *baicalensis*		Binds to 3CLpro active site	Vero E6 cells/CCK8 assays			33.0 at 100 μM	[126]
	Scutellarein (6-hydroxyapigenin)	*Scutellaria, Erigerontis herba*		Binds to the 3CLpro substrate binding site.	Vero cells/RT PCR	5.80 ± 0.22			[119]
		*Scutellaria* *baicalensis*		Binds to 3CLpro active site	Vero E6 cells/CCK8 assays	3.02 ± 0.11		101.6 at 100 µM 90.7 at 10 µM	[126]
	Scutellarin (scutellarein-7-glucuronide)	*Scutellaria, Erigerontis herba*		Binds to the 3CLpro substrate binding site.	Vero cells/RT PCR			28.9 ± 1.6 at 50 µM	[119]
		*Scutellaria* *baicalensis*		Binds to 3CLpro active site	Vero E6 cells/CCK8 assays			76.8 at 100 µM 18.9 at 10 µM	[126]
	Vitexin	Black garlic extract	IC50: 137 ± 10 µg/mL 100% inhibition at 0.5 mg/mL	Inhibits 3CLpro activity	FRET assay	180 ± 6		52 at 200 µM	[118]
	Wogonin	*Scutellaria* *baicalensis*	IC50: 8.52 ± 0.54 µg/mL EC50: 0.74 ± 0.36 µg/mL	Binds to the 3CLpro substrate binding site.	Vero cells/RT PCR			6.1 ± 0.8 at 50 µM	[119]
				Binds to 3CLpro active site	Vero E6 cells/CCK8 assays			3.6 at 100 µM	[126]
	Wogonoside	*Scutellaria* *baicalensis*	IC50: 8.52 ± 0.54 µg/mL EC50: 0.74 ± 0.36 µg/mL	Binds to the 3CLpro substrate binding site.	Vero cells/RT PCR			8.5 ± 3.3 at 50 µM	[119]
				Binds to 3CLpro active site	Vero E6 cells/CCK8 assays			20.4 at 100 µM	[126]
Isoflavones	Biochanin A			Interacts with 3CLpro catalytic site	FRET assay			5 at 10 µM	[117]
	Daidzein	Black garlic extract	IC50: 137 ± 10 µg/mL 100% inhibition at 0.5 mg/mL	Inhibits 3CLpro activity	FRET assay	56		100 at 200 µM	[118]
				Interacts with 3CLpro catalytic site	FRET assay			13.9 at 10 µM	[117]
	Formononetin			Interacts with 3CLpro catalytic site	FRET assay			16.0 at 10 µM	[117]
	Genistein			Interacts with 3CLpro catalytic site	FRET assay			15.0 at 10 µM	[117]
	Genistin	Black garlic extract	IC50: 137 ± 10 µg/mL 100% inhibition at 0.5 mg/mL	Inhibits 3CLpro activity	FRET assay			48 at 200 µM	[118]
				Interacts with 3CLpro catalytic site	FRET assay			25.5 at 10 µM	[117]
	Puerarin	Black garlic extract	IC50: 137 ± 10 µg/mL 100% inhibition at 0.5 mg/mL	Inhibits 3CLpro activity	FRET assay	42 ± 2		100 at 200 µM	[118]
	Sophoricoside			Interacts with 3CLpro catalytic site	FRET assay			10.3 at 10 µM	[117]
Flavan-3-ols/Flavanols	Catechin	Black garlic extract	IC50: 137 ± 10 µg/mL 100% inhibition at 0.5 mg/mL	Inhibits 3CLpro activity	FRET assay			9 at 200 µM	[118]
				Interacts with 3CLpro catalytic site	FRET assay			14.0 at 10 µM	[117]
	Catechin gallate (CG)	Black garlic extract	IC50: 137 ± 10 µg/mL 100% inhibition at 0.5 mg/mL	Inhibits 3CLpro activity	FRET assay			21 at 200 µM	[118]
	Epicatechin (EC)	Black garlic extract	IC50: 137 ± 10 µg/mL 100% inhibition at 0.5 mg/mL	Inhibits 3CLpro activity	FRET assay			8 at 200 µM	[118]
	Epicatechin gallate (ECG)	Black garlic extract	IC50: 137 ± 10 µg/mL 100% inhibition at 0.5 mg/mL	Inhibits 3CLpro activity	FRET assay			21 at 200 µM	[118]
	Epigallocatechin (EGC)	Black garlic extract	IC50: 137 ± 10 µg/mL 100% inhibition at 0.5 mg/mL	Inhibits 3CLpro activity	FRET assay			23 at 200 µM	[118]
	Epigallocatechin gallate (EGCG)	Black garlic extract	IC50: 137 ± 10 µg/mL 100% inhibition at 0.5 mg/mL	Inhibits 3CLpro activity	FRET assay	171 ± 5		53 at 200 µM	[118]
		TCM		Binds to the 3CLpro substrate binding site.	FRET assay	0.847 ± 0.005			[121]
	Gallocatechin gallate (GCG)	Black garlic extract	IC50: 137 ± 10 µg/mL 100% inhibition at 0.5 mg/mL	Inhibits 3CLpro activity	FRET assay			50 at 200 µM	[118]
Tannoid	Tannic acid	Black garlic extract	IC50: 137 ± 10 µg/mL, 100% inhibition at 0.5 mg/mL	Inhibits 3CLpro activity	FRET assay	9		100 at 200 µM	[118]
Others	Mixture of 11 flavonols	*Salvadora persica* L.		Inhibits 3CLpro activity	3CL protease assay, A549 cells	8.59 ± 0.3 μg mL^−1^		85.56 ± 1.12%	[127]

**Table 2:** Flavonoids with antiviral activities reported against SARS-CoV-2 PLpro using in vitro methodologies segregated according to class.

Class	Flavonoid	Natural Source (N.S.)/Extract	Mode of Action	Methods Used	% Inhibition	Reference
Flavonol	Myricetin		Interacts with 3CLpro catalytic site	FRET assay	50 at 159.10 ± 38.33 µM	[117]
	Rutin		Binds to naphthalene inhibitor binding pocket	PLpro enzymatic inhibition assay	38 at 100 µM	[128]
Flavones	Baicalein	*Scutellaria baicalensis*	Binds to 3CLpro active site	Vero E6 cells/CCK8 assays	45.1 at 50 µM, 12.4 at 12.5 µM	[126]
	Baicalin	*Scutellaria baicalensis*	Binds to 3CLpro active site	Vero E6 cells/CCK8 assays	15.9 at 50 µM	[126]
	Chrysin-7-O-β-D-glucuronide	*Scutellaria baicalensis*	Binds to 3CLpro active site	Vero E6 cells/CCK8 assays	16.3 at 50 µM	[126]
	Luteoloside (cyranoside)	*L. japonica*	Binds to 3CLpro active site	Vero E6 cells/CCK8 assays	21.5 at 50 μM	[126]
	Oroxylin A-7-O-β-D-glucuronide	*Scutellaria baicalensis*	Binds to 3CLpro active site	Vero E6 cells/CCK8 assays	7.4 at 50 μM	[126]
	Scutellarein	*Scutellaria baicalensis*	Binds to 3CLpro active site	Vero E6 cells/CCK8 assays	65.7 at 50 µM, 14.4 at 12.5 µM	[126]
	Scutellarin	*Scutellaria baicalensis*	Binds to 3CLpro active site	Vero E6 cells/CCK8 assays	41.1 at 50 µM, 12.7 at 12.5 µM	[126]
	Wogonin	*Scutellaria baicalensis*	Binds to 3CLpro active site	Vero E6 cells/CCK8 assays	52.0 at 50 µM, 35.9 at 12.5 µM	[126]
	Wogonoside	*Scutellaria baicalensis*	Binds to 3CLpro active site	Vero E6 cells/CCK8 assays	14.4 at 50 µM	[126]
Flavan-3-ols/Flavanols	Epicatechin gallate (ECG)		Binds to naphthalene inhibitor binding pocket	PLpro enzymatic inhibition assay	20 at 100 µM	[128]
	Epigallocatechin gallate (EGCG)		Binds to naphthalene inhibitor binding pocket	PLpro enzymatic inhibition assay	13 at 100 µM	[128]
Anthocyanin	Cyanidin-3-O-glucoside (chrysanthemin)		Binds to naphthalene inhibitor binding pocket	PLpro enzymatic inhibition assay	20 at 100 µM	[128]
Others	Hypericin		Binds to naphthalene inhibitor binding pocket	PLpro enzymatic inhibition assay	97 at 100 µ M87 at 50 µM	[128]

**Table 3:** Flavonoids with antiviral activities reported against SARS-CoV-2 spike protein–ACE2 interaction using in vitro methodologies segregated according to class.

Class	Flavonoid	Natural Source (N.S.)/Extract	Efficacy of N.S. Extract	Mode of Action	Methods Used	IC50 (µM)	% Inhibition	Reference
Flavonol	Isorhamnetin	Sea buckthorn berry		Binds to three residues involved in spike RBD–ACE2 interaction	HEK293 cells/SPR assay	Kd = 2.51 ± 0.68 μM		[125]
				Inhibits rhACE2 activity	MCA Fluorescence, rhACE2 cells		14.7 ± 1.4 at 10 µM	[129]
	Quercetin	*Hippophae rhamnoides L.*		Binds to three residues involved in spike RBD–ACE2 interaction	HEK293 cells/SPR assay	Kd = 5.92 ± 0.92 μM		[125]
				Inhibits rhACE2 activity	MCA Fluorescence, rhACE2 cells	At 2.5 min: 4.48 At 10.5 min: 29.5	66.2 ± 2.2 at 10 µM	[129]
	Quercetin-3-O-galactoside (hyperoside)			Inhibits rhACE2 activity	MCA Fluorescence, rhACE2 cells		34.2 ± 3.7 at 10 µM	[129]
	Quercetin-3-O-glucuronide			Inhibits rhACE2 activity	MCA Fluorescence, rhACE2 cells		33.1 ± 4.9 at 10 µM	[129]
	Quercetin-3-O-glucoside (isoquercetin)			Inhibits rhACE2 activity	MCA Fluorescence, rhACE2 cells		47.7 ± 3.7 at 10 µM	[129]
	Rutin (quercetin-3-)-rutinose)			Inhibits rhACE2 activity	MCA Fluorescence, rhACE2 cells		48.3 ± 4.7 at 10 µM	[129]
	Tamarixetin			Inhibits rhACE2 activity	MCA Fluorescence, rhACE2 cells		41.5 ± 5.0 at 10 µM	[129]
Flavanone	(±)-Eriodictyol			Inhibits rhACE2 activity	MCA Fluorescence, rhACE2 cells		24.4 ± 1.4 at 10 µM	[129]
	Hesperetin	*Anatolian Propolis*	IC50: 1.14 µL		S1 colorimetric assay	16,880		[130]
	Pinocembrin	*Anatolian Propolis*	IC50: 1.14 µL		S1 colorimetric assay	29,530		[130]
Flavones	Luteolin			Inhibits rhACE2 activity	MCA Fluorescence, rhACE2 cells		37.1 ± 0.6 at 10 µM	[129]
	Neobaicalein	*Radix Scutellariae*		Binds to ACE2 receptor	CMC, HEK293T cells, CK8 assay, SPR assay	83.8		[120]
	Oroxylin A	*Radix Scutellariae*		Binds to ACE2 receptor	CMC, HEK293T cells, CK8 assay, SPR assay	164.6		[120]
	Scutellarin	*Radix Scutellariae*		Binds to ACE2 receptor	CMC, HEK293T cells, CK8 assay, SPR assay	170.9		[120]
	Wogonin	*Radix Scutellariae*		Binds to ACE2 receptor	CMC, HEK293T cells, CK8 assay, SPR assay	137.6		[120]
Flavan-3-ols/Flavanols	Epicatechin (EC)	Green tea		Interferes with SARS-CoV-2 spike RBD–ACE2 interaction	HEK293T-ACE2 cells/Huh7 cells/Vero cells	>20 µg/mL		[131]
				Inhibits rhACE2 activity	MCA Fluorescence, rhACE2 cells		27.4 ± 5.7 at 10 µM	[129]
	Epigallocatechin gallate (EGCG)	Green tea		Interferes with SARS-CoV-2 spike RBD–ACE2 interaction	HEK293T-ACE2 cells/Huh7 cells/Vero cells/Plaque reduction assay	2.47 µg/mL		[131]
Anthocyanin	Pelargonidin			Binds to fatty acid binding pocket on spike RBD and attenuates spike–ACE2 interaction Reduces SARS-CoV-2 replication	ACE2-SARS-CoV-2 spike inhibitor screening assay Vero cells/Plaque assay		Screening Assay: >5 (10 µM), >15 (20 µM), >40 (50 µM)	[132]

**Table 4:** Flavonoids with antiviral activities reported against other SARS-CoV-2 targets using in vitro methodologies segregated according to class.

Target	Class	Flavonoid	Natural Source (N.S.)/Extract	Mode of Action	Methods Used	IC50 (µM)	EC50 (µM)	% Inhibition	Reference
RdRp	Flavone	Baicalein	*Scutellaria baicalensis*	Binding to NiRAN domain and the palm subdomain	Vero CCL-81/Calu-3 cells/MTS assay/qRT-PCR assay/293T cells/Huh7.5 cells		Vero: 4.5 ± 0.2 Calu-3: 1.2 + 0.03	99.8 at 20 µM	[134]
	Flavone	Baicalin	*Scutellaria baicalensis*	Inhibits RdRp	Vero CCL-81/Calu-3 cells/MTS assay/qRT-PCR assay/293T cells/Huh7.5 cells		Vero: 9.0 ± 0.08 Calu-3: 8.0 ± 0.11	98 at 20 µM	[134]
N	Flavanol/flavan-3-ol	Gallocatechin Gallate (GCG)	Green Tea	Disrupts the LLPS of N by interfering with N-RNA binding	H1299 cells RT-qPCR	44.4			[1]
Mitochondrial OXPHOS	Flavone	Baicalein	*Scutellaria baicalensis*	Oxygen consumption inhibitor	Vero E6 cells	10			[135]
nsP15	Flavanol/flavan-3-ol	epigallocatechin gallate (EGCG)	Green Tea extract	Binds to nsp15 active site	Endoribonuclease assay, plaque assay, Vero cells	1.62 ± 0.36	PRNT50: 0.2 µM		[136]
	Flavone	Baicalin	*Scutellaria baicalensis*, *Scutellaria lateriflora*		Endoribonuclease assay, plaque assay, Vero cells	7.98 ± 1.46	PRNT50: 83.3 µM		[136]
	Flavone	Baicalein	*Scutellaria baicalensis*, *Scutellaria lateriflora*		Endoribonuclease assay, plaque assay, Vero cells	8.61			[136]
	Flavonol	Quercetin	Onion peels, red grapes, green leafy vegetables		Endoribonuclease assay, plaque assay, Vero cells	13.79			[136]

**Table 5:** Some flavonoids and their natural source extracts are currently in clinical trials on COVID-19 patients. Extracted on 17 August 2021 from https://clinicaltrials.gov, accessed on 3 June 2021.

Study Title	Study Type	Number of Subjects Enrolled	Status
Nigella Sativa in COVID-19 (NCT04401202)	Prospective, Randomized, Open-label	183 COVID-19 positive participants *	Completed
Efficacy of Psidii Guava’s Extract For COVID-19 (NCT04810728)	Experimental, randomized, double-blind clinical trial	90 COVID-19 positive participants b/w 13-59 yrs.	Phase 3
The Effectiveness of Phytotherapy in SARS-CoV 2 (COVID-19) (NCT04851821)	Randomized, double—masked, interventional clinical Trial with Parallel Assignment	80 COVID-19 positive participants *	Phase 1
Masitinib Combined with Isoquercetin and Best Supportive Care in Hospitalized Patients With Moderate and Severe COVID-19 (NCT04622865)	Randomized, double-blinded, triple-masked interventional clinical trial with Parallel Assignment	200 COVID-19 positive participants *	Phase 2
Quercetin In The Treatment of SARS-CoV 2 (NCT04853199)	Randomized, double-blinded, triple-masked interventional clinical trial with Parallel Assignment	200 COVID-19 positive participants *	Early Phase 1
Randomized Proof-of-Concept Trial to Evaluate the Safety and Explore the Effectiveness of Resveratrol, a Plant Polyphenol, for COVID-19 (NCT04400890)	Randomized placebo-controlled, double-blinded, quadruple-masked, interventional clinical trial	100 COVID-19 positive participants ≥ 45 yrs.	Phase 2
Tannin Specific Natural Extract for COVID-19 Infection (NCT04403646)	Double-blind, randomized, triple-masked	124 COVID-19 positive participants *	n/a
P2Et Extract in the Symptomatic Treatment of Subjects With COVID-19 (NCT04410510)	Double-blind, randomized, triple-masked, interventional clinical trial with parallel assignment	100 COVID-19 positive participants *	Phase 2/3
COVID-19, Hospitalized, Patients, Nasafytol (NCT04844658)	Standard-of-care comparative, open-labelled, parallel two-arms and randomized trial	50 COVID-19 positive participants *	Recruiting
Study to Investigate the Clinical Benefits of Dietary Supplement Quercetin for Managing Early COVID-19 Symptoms at Home (NCT04861298)	Open-labelled, randomized, parallel-assignment, interventional trial	142 COVID-19 positive participants *	Recruiting
Complementary Intervention for COVID-19 (NCT04487964)	Open-labelled, non-randomized	70 COVID-19 positive participants *	Recruiting
The Study of Quadruple Therapy Zinc, Quercetin, Bromelain and Vitamin C on the Clinical Outcomes of Patients Infected With COVID-19 (NCT04468139)	Open-labelled, single-assignment, interventional trial	60 COVID-19 positive participants *	Phase 4
Evaluation of the Effect of Anatolian Propolis on COVID-19 in Healthcare Professionals (NCT04680819)	Observational, prospective cohort study	50 HCWs at risk for developing COVID-19	Not yet recruiting

NLR: Neutrophil to Lymphocyte Ratio; hs-CRP: high-sensitivity C-reactive protein; ARDS: acute respiratory distress syndrome; PDI: Protein disulfide-isomerase; * participants ≥ 18 yrs.

## Data Availability

No new data were created or analyzed in this study.

## Supplementary Materials

The following are available online at https://www.mdpi.com/article/10.3390/ijms222011069/s1.
