# Supplementary material for: Promising Antiviral Activities of Natural Flavonoids against SARS-CoV-2 Targets: Systematic Review
Source: Int J Mol Sci. 2021 Oct 14;22(20):11069. doi: 10.3390/ijms222011069 (PMC8539743; doi:10.3390/ijms222011069)
Supplement: Supplementary file 1 [file ijms-22-11069-s001.zip › ijms-1390175-supplementary.pdf]

Supplementary material:

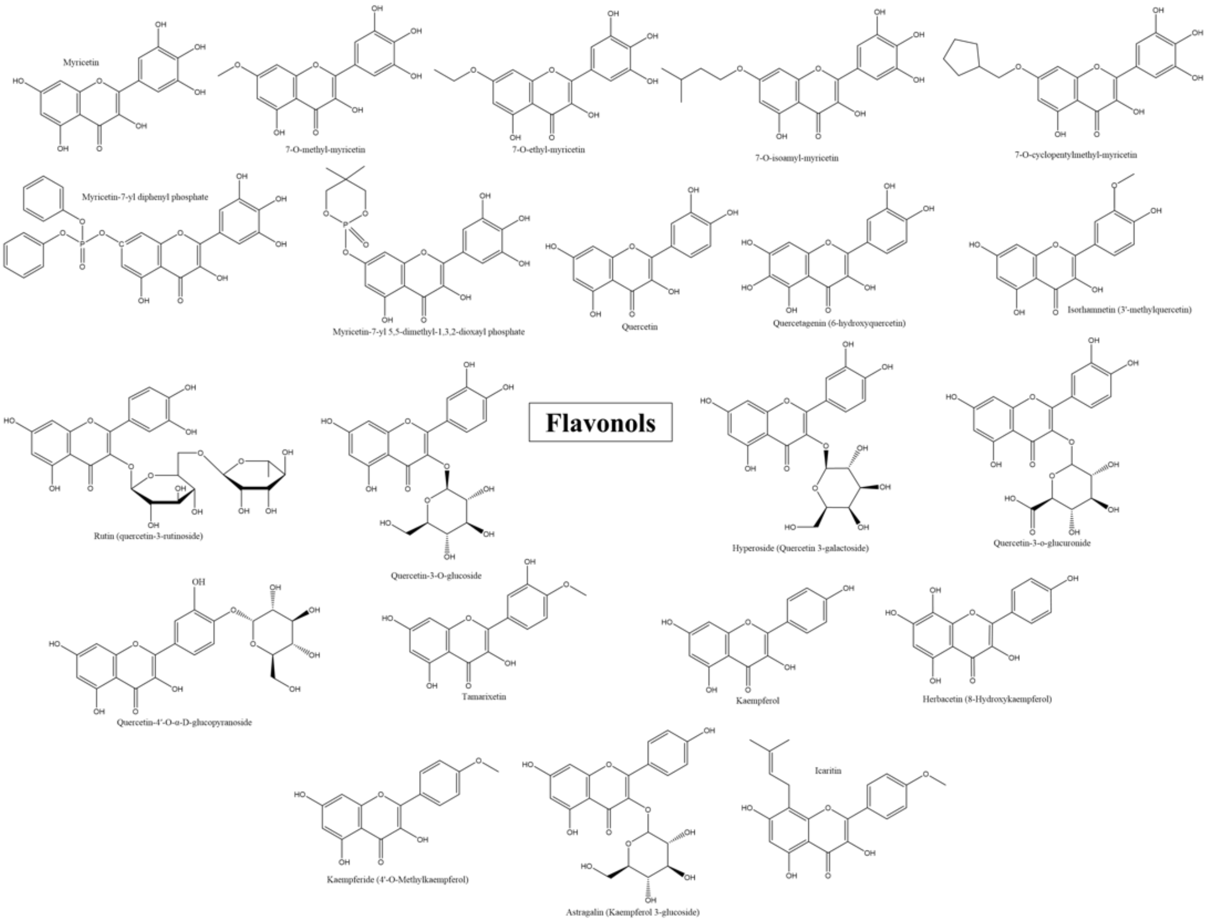

Figure S1. Flavonols reported to have anti-SARS-CoV-2 activities; images adapted from PubChem (https://pubchem.ncbi.nlm.nih.gov, accessed on 3 June 2021) and created using ChemDraw (https://perkinelmerinformatics.com/products/research/chemdraw/, accessed on 3 June 2021).

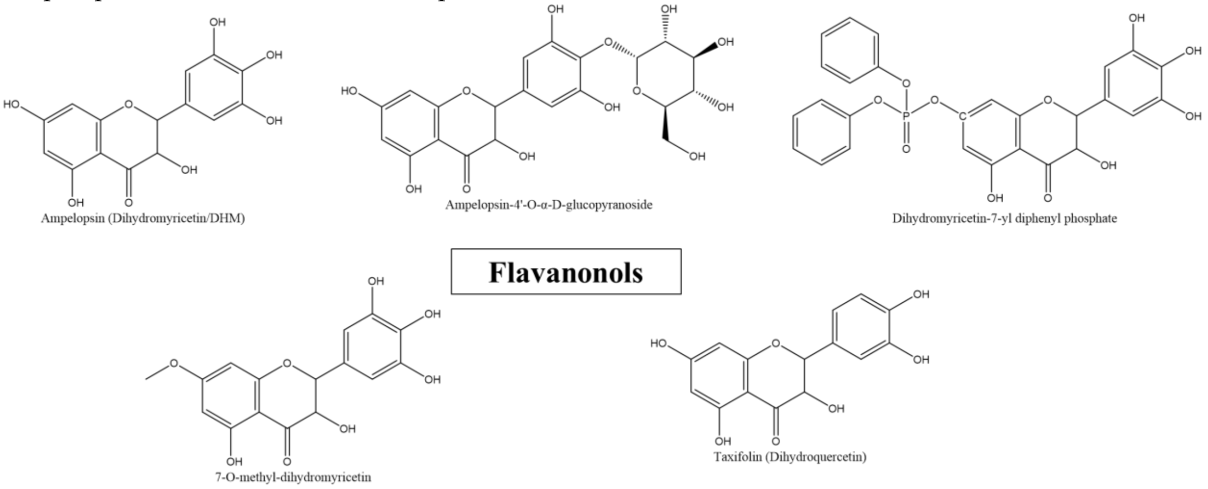

Figure S2. Flavanonols reported to have anti-SARS-CoV-2 activities; images adapted from PubChem (https://pubchem.ncbi.nlm.nih.gov, accessed on 3 June 2021) and created using ChemDraw (https://perkinelmerinformatics.com/products/research/chemdraw/, accessed on 3 June 2021).

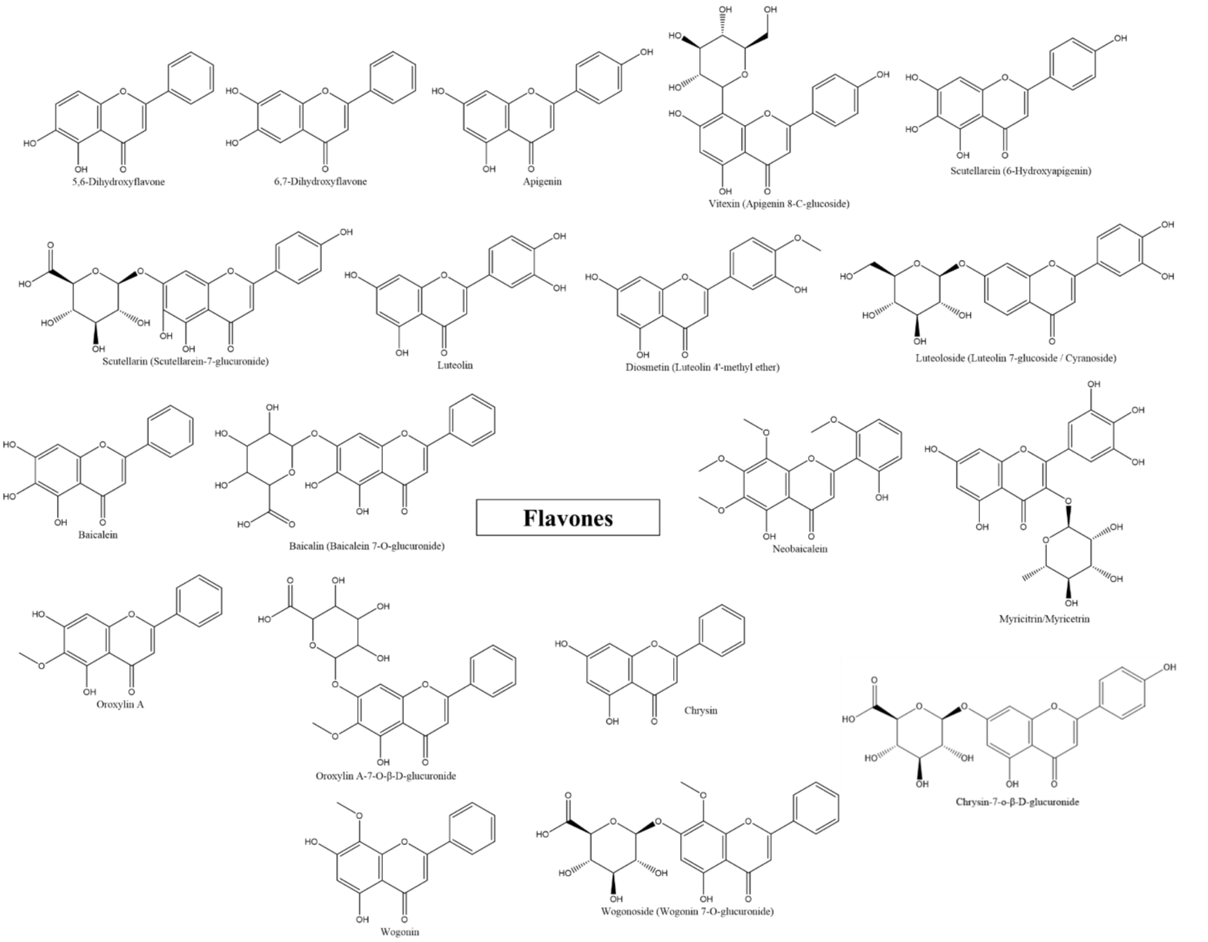

Figure S3. Flavones reported to have anti-SARS-CoV-2 activities; images adapted from PubChem (https://pubchem.ncbi.nlm.nih.gov, accessed on 3 June 2021) and created using ChemDraw (https://perkinelmerinformatics.com/products/research/chemdraw/, accessed on 3 June 2021).

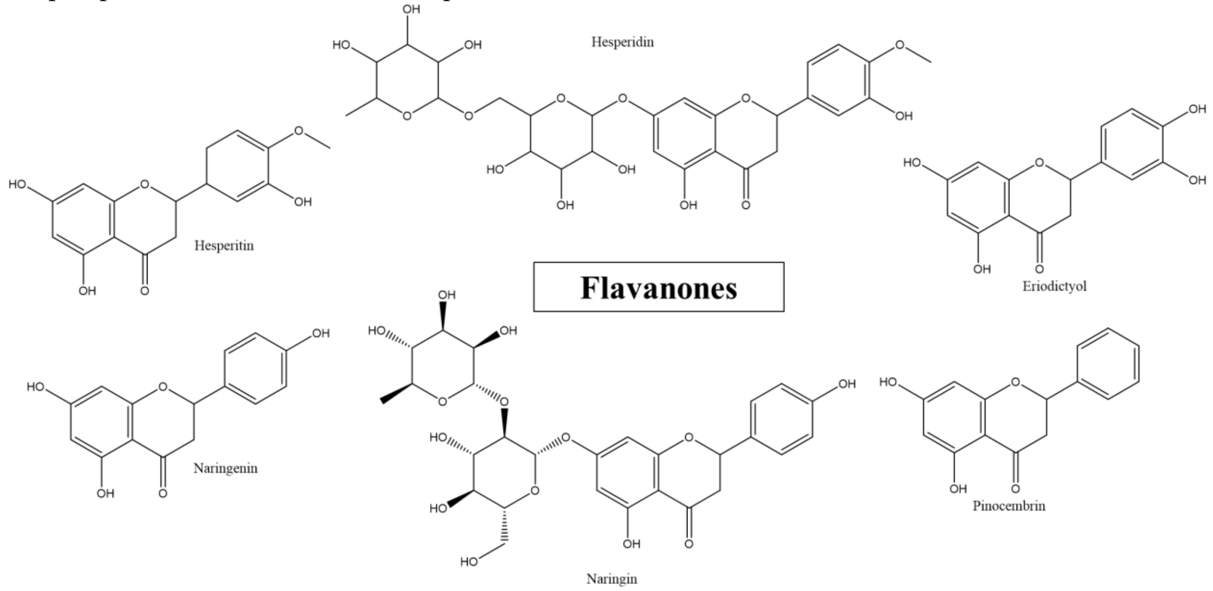

Figure S4. Flavanones reported to have anti-SARS-CoV-2 activities; images adapted from PubChem (https://pubchem.ncbi.nlm.nih.gov, accessed on 3 June 2021) and created using ChemDraw (https://perkinelmerinformatics.com/products/research/chemdraw/, accessed on 3 June 2021).

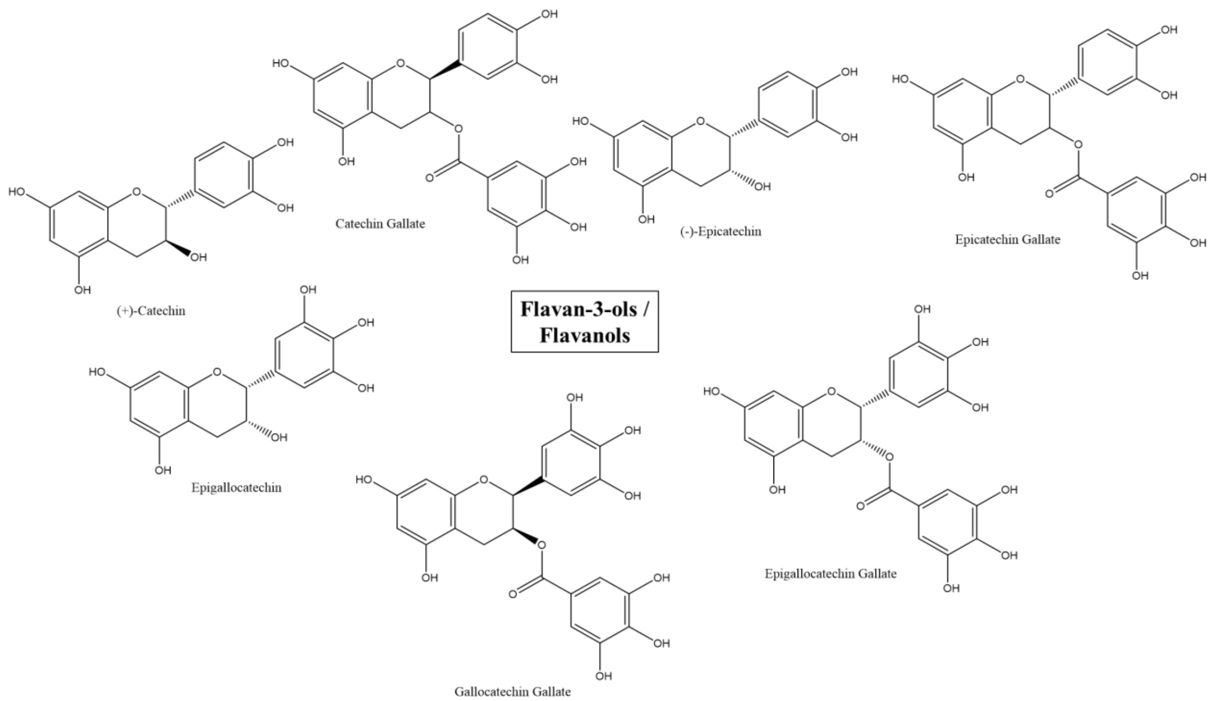

Figure S5. Flavan-3-ols / Flavanols reported to have anti-SARS-CoV-2 activities; images adapted from PubChem (https://pubchem.ncbi.nlm.nih.gov, accessed on 3 June 2021) and created using ChemDraw (https://perkinelmerinformatics.com/products/research/chemdraw/, accessed on 3 June 2021).

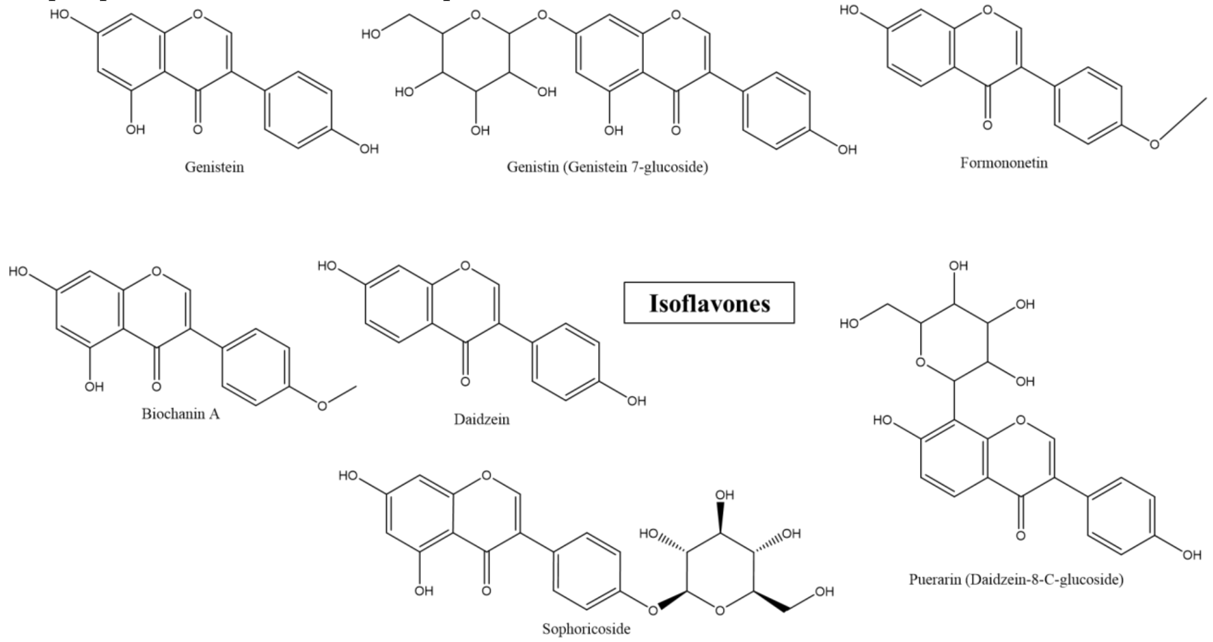

Figure S6. Isoflavones reported to have anti-SARS-CoV-2 activities; images adapted from PubChem (https://pubchem.ncbi.nlm.nih.gov, accessed on 3 June 2021) and created using ChemDraw (https://perkinelmerinformatics.com/products/research/chemdraw/, accessed on 3 June 2021).

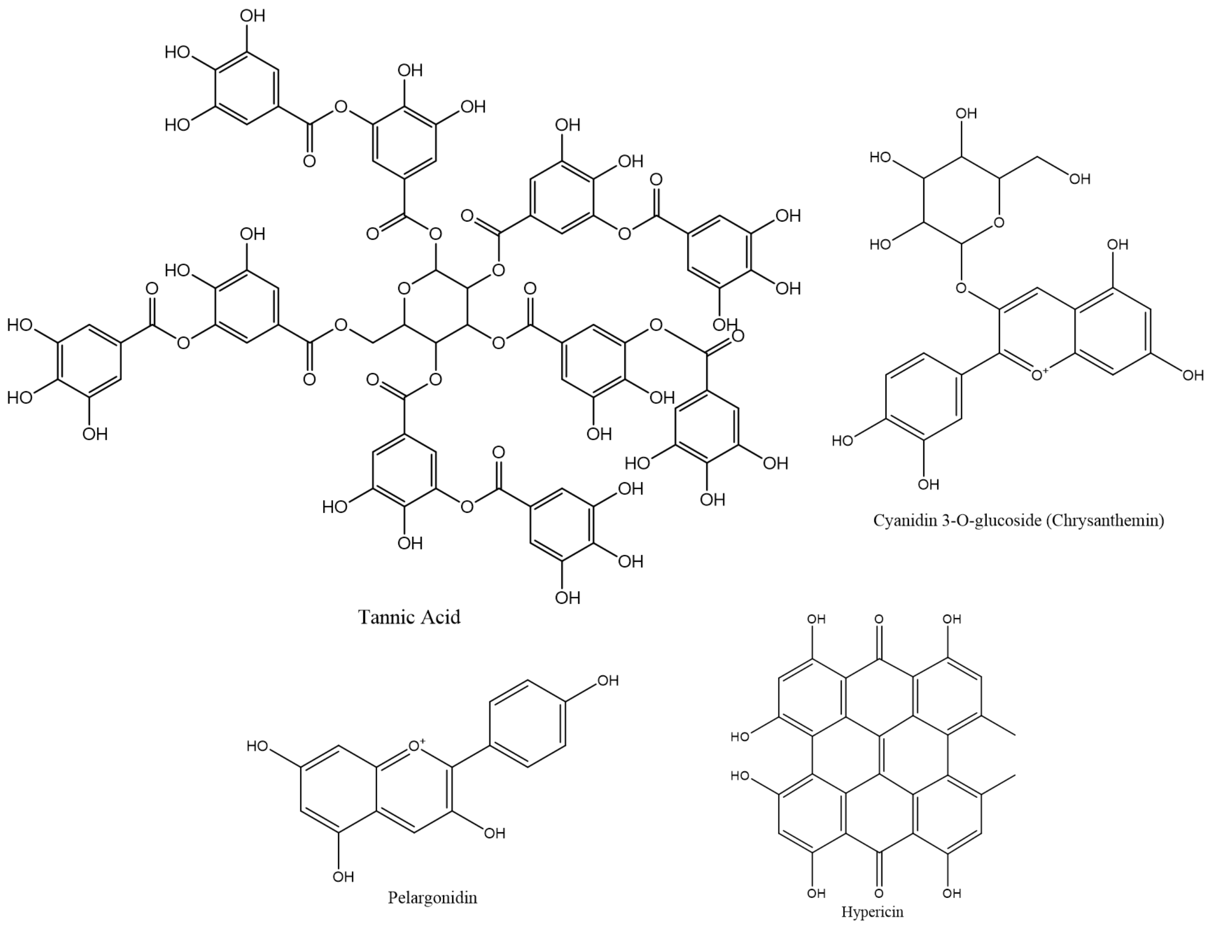

Figure S7. Other flavonoid-related compounds reported to have anti-SARS-CoV-2 activities; images adapted from PubChem (https://pubchem.ncbi.nlm.nih.gov, accessed on 3 June 2021) and created using ChemDraw (https://perkinelmerinformatics.com/products/research/chemdraw/, accessed on 3 June 2021).

Table S1. Flavonoids with antiviral activities reported against animal and human coronaviruses, including SARS-CoV and MERS-CoV, using *in vitro* methodologies segregated according to virus and target.

Viral Target	Class	Flavonoid(s)	Natural Source	Methods used	Efficacy	Reference
Bovine Coronavirus	Flavone	Chrysin	Georgian "bouleaue" propolis	Plaque reduction assay	45% inhibition of replication at 10 μg/ml	[76]
	Flavonol	Kaempferol	Georgian "bouleaue" propolis	Plaque reduction assay	65% inhibition of replication at 10 μg/ml	[76]
	Flavonol	Quercetin	Georgian "bouleaue" propolis	Plaque reduction assay	~50% inhibition of infectivity at 60 μg/ml	[76]
	Theaflavin	Theaflavin, theaflavin-3-monogallate, theaflavin-3'-monogallate and theaflavin-3,3' digallate	Black tea	Neutralization or infectivity assays using BSC-1 cells and HRT cells	EC50 = 34.7 ± 9 μg/ml	[106]
Porcine epidemic diarrhea virus (PEDV)	Flavonol	Quercetin 7-rhamnoside	*Houttuynia cordata*	Sulforhodamine B (SRB) assay using Vero cells	IC50 = 0.014 ± 0.005 μg/mL, 85.1% inhibition of infectivity at 10 μg/mL	[107]
	Flavonol	Quercetin	*Houttuynia cordata*	Sulforhodamine B (SRB) assay using Vero cells	IC50 = 1.7 ± 0.8 μg/mL	[107]
	Flavone	Apigenin	*Houttuynia cordata*	Sulforhodamine B (SRB) assay using Vero cells	IC50 = 0.1 ± 0.1 μg/mL	[107]
	Flavone	Luteolin	*Houttuynia cordata*	Sulforhodamine B (SRB) assay using Vero cells	IC50 = 0.2 ± 0.2 μg/mL	[107]
	Flavan-3-ol / Flavanol	Catechin	*Houttuynia cordata*	Sulforhodamine B (SRB) assay using Vero cells	IC50 = 11.1 ± 7.1 μg/mL	[107]
Porcine respiratory coronavirus (PRCV)	Flavonol	Quercetin 7-rhamnoside	*Houttuynia cordata*	Sulforhodamine B (SRB) assay using ST cells	IC50 = 59.76 ± 5.70 μg/mL	[107]
Human Coronavirus OC43	Flavone	Chrysin	Georgian "bouleaue" propolis	Plaque reduction assay	30% inhibition of replication at 10 μg/ml	[76]
	Flavonol	Kaempferol	Georgian "bouleaue" propolis	Plaque reduction assay	50% inhibition of replication at 10 μg/ml	[76]
	Flavonol	Quercetin	Georgian "bouleaue" propolis	Plaque reduction assay	~50% inhibition of infectivity at 60 μg/ml	[76]
SARS-CoV-1 3CLpro	Biflavone	Amentoflavone	*Torreya nucifera* leaves	FRET assay	IC50 = 8.3 μM	[108]
	Biflavone	bilobetin)	*Torreya nucifera* leaves	FRET assay	IC50 = 72.3 ± 4.5 μM, Ki = 13.8 ± 1.5 μM (Non-competitive)	[108]
	Biflavone	Ginkgetin	*Torreya nucifera* leaves	FRET assay	IC50 = 32.0 ± 1.7 μM, Ki = 80.4 ± 4.0 μM (Non-competitive)	[108]

	Biflavone	Sciadopitysin	*Torreya nucifera* leaves	FRET assay	IC50 = 38.4 ± 0.2 µM, Ki = 30.2 ± 2.6 µM (Non-competitive)	[108]
	Flavone	Apigenin		FRET assay	IC50 = 280.8 µM, Ki = 35.6 ± 1.1 µM (Non-competitive)	[108]
	Flavone	Luteolin		FRET assay	IC50 = 20.2 µM	[108]
	Flavonol	Quercetin		FRET assay	IC50 = 23.8 µM	[108]
	Flavonol	Herbacetin		FRET assay	IC50 = 33.17 µM	[109]
	Flavone	Rhoifolin		FRET assay	IC50 = 27.45 µM	[109]
	Flavone	Pectolinarin		FRET assay	IC50 = 37.78 µM	[109]
	Flavonol	Quercetin	*Pichia pastoris*	FRET assay	IC50 = 73 ± 4 µM, 82% inhibition at 200 µM	[110]
	Isoflavone	Puerarin	*Pichia pastoris*	FRET assay	IC50 = 381 ± 12.5 µM, 33% inhibition at 200 µM	[110]
	Isoflavone	Daidzein	*Pichia pastoris*	FRET assay	IC50 = 351 ± 2.9 µM, 34% inhibition at 200 µM	[110]
	Flavan-3-ol / Flavanol	EGC	*Pichia pastoris*	FRET assay	5.4% inhibition at 200 µM	[110]
	Flavan-3-ol / Flavanol	EGCG	*Pichia pastoris*	FRET assay	IC50 = 73 ± 2 µM, 85% inhibition at 200 µM	[110]
	Flavan-3-ol / Flavanol	GCG	*Pichia pastoris*	FRET assay	IC50 = 47 ± 0.9 µM, 91% inhibition at 200 µM, Ki = 25 ± 1.7 µM (Competitive)	[110]
	Chalcone	Broussochalcone B	*Broussonetia papyrifera*	FRET assay	IC50 = 57.8 ± 0.5 µM	[111]
	Chalcone	Broussochalcone A	*Broussonetia papyrifera*	FRET assay	IC50 = 88.1 ± 13.0 µM	[111]
	Chalcone	4-hydroxyisolonchocarpin	*Broussonetia papyrifera*	FRET assay	IC50 = 202.7 ± 3.9 µM	[111]
	Flavonol	Papyriflavonol A	*Broussonetia papyrifera*	FRET assay	IC50 = 103.6 ± 17.4 µM	[111]
	Flavan	3 ′ - (3-methylbut-2-enyl) - 3 ′, 4,7-trihydroxyflavan	*Broussonetia papyrifera*	FRET assay	IC50 = 30.2 ± 6.8 µM	[111]
	Flavan	Kazinol A	*Broussonetia papyrifera*	FRET assay	IC50 = 84.8 ± 10.4 µM	[111]
	Flavan	Kaznol B	*Broussonetia papyrifera*	FRET assay	IC50 = 233.3 ± 6.7 µM	[111]
	Flavan	Broussoflavan A	*Broussonetia papyrifera*	FRET assay	IC50 = 92.4 ± 2.1 µM	[111]
	Chalcone	Isoliquiritigenin		FRET assay	IC50 = 61.9 ± 11.0 µM	[111]
	Flavonol	Kaempferol		FRET assay	IC50 = 116.3 ± 7.1 µM	[111]

	Flavonol	Quercetin		FRET assay	IC50 = 52.7 ± 4.1 µM	[111]
	Flavonol	Quercetin-β-galactoside		FRET assay	IC50 = 128.8 ± 4.5 µM	[111]
SARS-CoV-1 PLpro	Novel geranylated flavanone	Tomentin A	*Paulownia tomentosa*	Fluorogenic assay	IC50 = 6.2 ± 0.04 µM, Ki = 4.8 µM (Mixed)	[112]
	Novel geranylated flavanone	Tomentin B	*Paulownia tomentosa*	Fluorogenic assay	IC50 = 6.1 ± 0.02 µM, Ki = 3.5 µM (Mixed)	[112]
	Novel geranylated flavanone	Tomentin C	*Paulownia tomentosa*	Fluorogenic assay	IC50 = 11.6 ± 0.13 µM, Ki = 5.0 µM (Mixed)	[112]
	Novel geranylated flavanone	Tomentin D	*Paulownia tomentosa*	Fluorogenic assay	IC50 = 12.5 ± 0.22 µM, Ki = 3.7 µM (Mixed)	[112]
	Novel geranylated flavanonol	Tomentin E	*Paulownia tomentosa*	Fluorogenic assay	IC50 = 5.0 ± 0.06 µM, Ki = 4.8 µM (Mixed)	[112]
	Geranylated flavanonol	3′-*O*-Methyldiplacol	*Paulownia tomentosa*	Fluorogenic assay	IC50 = 9.5 ± 0.10 µM, Ki = 6.6 µM (Mixed)	[112]
	Geranylated flavanonol	4′-*O*-Methyldiplacol	*Paulownia tomentosa*	Fluorogenic assay	IC50 = 9.2 ± 0.13 µM, Ki = 6.3 µM (Mixed)	[112]
	Geranylated flavanone	3′-*O*-Methyldiplacone	*Paulownia tomentosa*	Fluorogenic assay	IC50 = 13.2 ± 0.14 µM, Ki = 7.1 µM (Mixed)	[112]
	Geranylated flavanone	4′-*O*-Methyldiplacone	*Paulownia tomentosa*	Fluorogenic assay	IC50 = 12.7 ± 0.19 µM, Ki = 6.9 µM (Mixed)	[112]
	Geranylated flavanone	Mimulone	*Paulownia tomentosa*	Fluorogenic assay	IC50 = 14.4 ± 0.27 µM, Ki = 7.8 µM (Mixed)	[112]
	Geranylated flavanone	Diplacone	*Paulownia tomentosa*	Fluorogenic assay	IC50 = 10.4 ± 0.16 µM, Ki = 5.1 µM (Mixed)	[112]
	Geranylated flavanone	6-Geranyl-4′,5,7-trihydroxy-3′,5′-dimethoxyflavanone	*Paulownia tomentosa*	Fluorogenic assay	IC50 = 13.9 ± 0.18 µM, Ki = 8.4 µM (Mixed)	[112]
	Chalcone	Broussochalcone B	*Broussonetia papyrifera*	FRET assay	IC50 = 11.6 ± 0.7 µM, Ki = 6.6 ± 0.5 µM (Non-competitive)	[111]
	Chalcone	Broussochalcone A	*Broussonetia papyrifera*	FRET assay	IC50 = 9.2 ± 1.5 µM, Ki = 8.0 ± 0.4 µM (Non-competitive)	[111]
	Chalcone	4-hydroxyisolonchocarpin	*Broussonetia papyrifera*	FRET assay	IC50 = 35.4 ± 11.3 µM, Ki = 27.7 ± 1.7 µM (Non-competitive)	[111]
	Flavonol	Papyriflavonol A	*Broussonetia papyrifera*	FRET assay	IC50 = 3.7 ± 1.6 µM, Ki = 5.9 ± 0.4 µM (Non-competitive)	[111]
	Flavan	3 ′ - (3-methylbut-2-enyl) - 3 ′, 4,7-trihydroxyflavan	*Broussonetia papyrifera*	FRET assay	IC50 = 35.8 ± 6.7 µM, Ki = 15.9 ± 0.8 µM (Non-competitive)	[111]

	Flavan	Kazinol A	*Broussonetia papyrifera*	FRET assay	IC50 = 66.2 ± 6.8 µM, Ki = 40.5 ± 3.4µM (Non-competitive)	[111]
	Flavan	Kaznol B	*Broussonetia papyrifera*	FRET assay	IC50 = 31.4 ± 2.9 µM, Ki = 36.7 ± 2.7 µM (Non-competitive)	[111]
	Flavan	Broussoflavan A	*Broussonetia papyrifera*	FRET assay	IC50 = 30.4 ± 5.5 µM, Ki = 23.4 ± 1.6 µM (Non-competitive)	[111]
	Chalcone	Isoliquiritigenin		FRET assay	IC50 = 24.6 ± 1.0 µM, Ki = 3.0 ± 0.9 µM (Non-competitive)	[111]
	Flavonol	Kaempferol		FRET assay	IC50 = 16.3 ± 2.1 µM, Ki = 13.7 ± 0.8µM (Non-competitive)	[111]
	Flavonol	Quercetin		FRET assay	IC50 = 8.6 ± 3.2 µM, Ki = 7.0 ± 0.7 µM (Non-competitive)	[111]
	Flavonol	Quercetin-β-galactoside		FRET assay	IC50 = 51.9 ± 5.5 µM, Ki = 56.1 ± 2.5µM (Non-competitive)	[111]
SARS-CoV-1 3a ion channel	Flavonol	Juglanin		*Xenopus* oocytes and voltage clamp methods	IC50 = 2.3 µM	[113]
SARS-CoV-1 helicase (nsP13)	Flavonol	Myricetin		Colorimetry-based ATP hydrolysis assay	IC50 = 2.71 ± 0.19 µM, >90% inhibition of ATPase activity at 10 µM	[114]
	Flavone	Scutellarein	*Scutettaria baicalensis*	Colorimetry-based ATP hydrolysis assay	IC50 = 0.86 ± 0.48 µM, >90% inhibition of ATPase activity at 10 µM	[114]
	Flavone	Myricitrin		Colorimetry-based ATP hydrolysis assay	~20% inhibition of ATPase activity at 10 µM	[114]
	Biflavone	Amentoflavone		Colorimetry-based ATP hydrolysis assay	~20% inhibition of ATPase activity at 10 µM	[114]
	Flavone	Diosmetin-7-*O*-Glc-Xyl	*Phseudolysimachion longifoiium*	Colorimetry-based ATP hydrolysis assay	~20% inhibition of ATPase activity at 10 µM	[114]
SARS-CoV-1 Spike-ACE2 interaction	Anthraquinone	Emodin	*Rheum* and *Polygonum*	S protein-pseudo typed retrovirus, Vero cells and MTT assay	IC50 = 200 µM against S-ACE2 interaction, 94.12 ± 5.90% inhibition of infectivity at 50 µM	[115]
SARS-CoV-1 (unspecified)	Flavone	Baicalin	*Scutellaria baicalensis*	Plaque reduction assay, neutralization test, fRhK4 cell and Vero cell	EC50 = 12.5 – 25 µg/ml at 48 h and 25-50 µg/ml at 72 h	[116]
MERS-CoV 3CLpro	Chalcone	Broussochalcone B	*Broussonetia papyrifera*	FRET assay	IC50 = 27.9 ± 1.2 µM	[111]

	Chalcone	Broussochalcone A	*Broussonetia papyrifera*	FRET assay	IC50 = 36.2 ± 0.4 μM	[111]
	Chalcone	4-hydroxyisolonchocarpin	*Broussonetia papyrifera*	FRET assay	IC50 = 193.7 ± 15.6 μM	[111]
	Flavonol	Papyriflavonol A	*Broussonetia papyrifera*	FRET assay	IC50 = 64.5 ± 4.9 μM	[111]
	Flavan	3 ′ - (3-methylbut-2-enyl) - 3 ′, 4,7-trihydroxyflavan	*Broussonetia papyrifera*	FRET assay	IC50 = 34.7 ± 2.0 μM	[111]
	Flavan	Broussoflavan A	*Broussonetia papyrifera*	FRET assay	IC50 = 125.7 ± 17.4 μM	[111]
	Chalcone	Isoliquiritigenin		FRET assay	IC50 = 33.9 ± 7.7 μM	[111]
	Flavonol	Kaempferol		FRET assay	IC50 = 35.3 ± 5.3 μM	[111]
	Flavonol	Quercetin		FRET assay	IC50 = 34.8 ± 1.2 μM	[111]
	Flavonol	Quercetin-β-galactoside		FRET assay	IC50 = 68.0 ± 2.4 μM	[111]
	Flavonol	Herbacetin		FRET assay	IC50 = 40.59 μM	[117]
	Chalcone	Isobavachalcone		FRET assay	IC50 = 35.85 μM	[117]
	Flavonol	Quercetin-3- β-D-glucoside		FRET assay	IC50 = 37.03 μM	[117]
	Chalcone	Helichrysetin		FRET assay	IC50 = 67.04 μM	[117]
MERS-CoV PLpro	Chalcone	Broussochalcone B	*Broussonetia papyrifera*	FRET assay	IC50 = 112.9 ± 10.1 μM	[111]
	Chalcone	Broussochalcone A	*Broussonetia papyrifera*	FRET assay	IC50 = 42.1 ± 5.0 μM	[111]
	Chalcone	4-hydroxyisolonchocarpin	*Broussonetia papyrifera*	FRET assay	IC50 = 171.6 ± 10.2 μM	[111]
	Flavonol	Papyriflavonol A	*Broussonetia papyrifera*	FRET assay	IC50 = 112.5 ± 7. μM	[111]
	Flavan	3 ′ - (3-methylbut-2-enyl) - 3 ′, 4,7-trihydroxyflavan	*Broussonetia papyrifera*	FRET assay	IC50 = 48.8 ± 6.6 μM	[111]
	Flavan	Kazinol A	*Broussonetia papyrifera*	FRET assay	IC50 = 88.5 ± 3.9 μM	[111]
	Flavan	Kaznol B	*Broussonetia papyrifera*	FRET assay	IC50 = 94.9 ± 13.1 μM	[111]
	Flavan	Broussoflavan A	*Broussonetia papyrifera*	FRET assay	IC50 = 49.1 ± 7.5 μM	[111]
	Chalcone	Isoliquiritigenin		FRET assay	IC50 = 82.2 ± 7.7 μM	[111]
	Flavonol	Kaempferol		FRET assay	IC50 = 206.6 ± 1.7 μM	[111]
	Flavonol	Quercetin-β-galactoside		FRET assay	IC50 = 129.4 ± 14.5 μM	[111]

Table S2. Some flavonoids and their natural source extracts are currently in clinical trials on COVID-19 patients. Extracted on 17 August 2021 from https://clinicaltrials.gov, accessed on 3 June 2021.

NTC #	Study Title	Study Type	Background	Hypothesis / Result	# of subjects enrolled	Polyphenol (s)	Treatment period	Treatment Dose	Status
NCT04401202	Nigella Sativa in COVID-19	Prospective, Randomized, Open-label, Controlled Clinical Study with parallel assignment	Natural products with immunomodulation and antiviral activity showed a promising improvement in the outcomes of some viral infectious diseases both in preclinical and primitive clinical studies.	To utilize the Saudi FDA licensed *Nigella sativa* seed oil towards improving disease outcomes in adult patients diagnosed with mild COVID-19.	183 COVID-19 positive participants ≥ 18 yrs.	Kaempferol and Quercetin [179]	10 days	*Nigella sativa* oil 500mg soft gel capsules in oral twice daily dose.<br><br>Control: no intervention	Completed<br><br>54/87 (62.1%) in the intervention group vs. 31/86 (36.0%) in the placebo group clinically recovered Within 14 Days After Randomization.<br><br>Mean number of Days until recovery: 11 (8 to 14) in intervention group vs. 14 (11 to 14) in control.
NCT04810728	Efficacy of Psidii Guava's Extract For COVID-19	Experimental, randomized, double-blind clinical trial, with a parallel design	Brazilian guava leaf, *Psidium Geunesse*, extract and its flavonoids (quercetin as the most promising) were found to show antiviral activities against DENV-2 and HIV-1; in particular against reverse transcriptase, RNA polymerase, protease, helicase and ATPase.	*Psidii* guava extract is hypothesized to improve WBC (neutrophil, lymphocyte, monocyte), NLR and hs-CRP levels; also hypothesized to increase proportion and shorten the duration of COVID-19 seroconversion in mild and	90 COVID-19 positive participants b/w 13-59 yrs.	Largely quercetin	7/28 days based on interim swab test result.	2 Capsules of extract Psidii guava, three times daily.<br>Control: standard therapy for Covid-19 patient:<br>(Vitamin C, Zinc, medication for clinical symptoms such as: antipyretic agent, decongestan and mucolytic.)	Phase 3

				symptomless cases.					
NCT04851821	The Effectiveness of Phytotherapy in SARS-COV2(COVID-19)	Randomized, double - masked, interventional clinical Trial with Parallel Assignment	Several medicinal plants, like ginkgo and St. John's Wort, owe part of their therapeutic effects to flavonoids like quercetin and rutin. When combined with supplementary Vitamin C, it improves absorption by the body and delays its elimination.	To establish whether Quercetin will have therapeutic relief on COVID-19 patients by measuring the efficacy of patients consulting the ED.	80 COVID-19 positive participants ≥ 18 yrs.	Quercetin	10 days	One 'Quercetix' tablet three times per day 30 minutes before a meal.<br><br>Placebo given to another group.	Phase 1
NCT04622865	Masitinib Combined With Isoquercetin and Best Supportive Care in Hospitalized Patients With Moderate and Severe COVID-19	Randomized, double-blinded, triple-masked interventional clinical trial with Parallel Assignment	Often patients with moderate and severe COVID-19, develop cytokine storms that leads to severe pulmonary inflammation and various thrombotic events associated with ARDS and may be fatal. Given that masitinib is a potent blocker of mast cells and macrophages that are contributors to the cytokine storm and that isoquercetin inhibits PDI, an enzyme directly involved in the formation of clots, and also decreases D-Dimer, a predictor of COVID-19 thrombosis severity, their combination may prevent the development of these two complications.	To evaluate efficacy of the masitinib and isoquercetin combination in moderate and severe COVID-19 patients	200 COVID-19 positive participants ≥ 18 yrs.	Isoquercetin	15 days	Oral masitinib dose of 3 mg/kg/day for 4 days, then 4.5 mg/kg/day.<br><br>Oral isoquercetin dose of 1 g/day.<br><br>Control: presence of comparable supportive care with no such intervention as above.	Phase 2
NCT04853199	Quercetin In The Treatment Of SARS-COV 2	Randomized, double-blinded, triple-masked interventional clinical trial with Parallel Assignment	Quercetin's antioxidant, anti-inflammatory, and antihistamine (antiallergic) properties have been observed in numerous *in vitro* and animal studies. Their effectiveness in reducing the permeability of blood vessels has also been noted.	Evaluation of Effectiveness of Quercetin in the treatment of SARS-COV-2 induced COVID-19	200 COVID-19 positive participants ≥ 18 yrs.	Quercetin	10 days	One Quercetin tablet twice a day 30 minutes before the meal.<br><br>Placebo capsule contains 150 mg of microcrystalline cellulose and 1.5 mg of magnesium stearate.	Early Phase 1

NCT04400890	Randomized Proof-of-Concept Trial to Evaluate the Safety and Explore the Effectiveness of Resveratrol, a Plant Polyphenol, for COVID-19	Randomized placebo-controlled, double-blinded, quadruple-masked, interventional clinical trial with parallel assignment.	Published *in vitro* data supports that this polyphenol inhibits coronavirus replication while separately published in silico (computer molecular docking analysis) reports have identified specific molecular targets of resveratrol against SARS-CoV2. Animal studies also demonstrate that resveratrol is effective at preventing lung injury and death in certain animal models of viral infections. Furthermore, the products long history as an anti-inflammatory might prevent the cytokine storm that is associated with worse outcomes in COVID-19.	Comparison of the effects of resveratrol to placebo to assess the safety of the resveratrol and explore effectiveness. The primary outcome measure for this trial is reduction in hospitalization at 21 days from enrollment.	100 COVID-19 positive participants ≥ 45 yrs.	Resveratrol	7-15 days depending on duration of symptoms.	Intervention Group: Resveratrol 1000mg four times per day for 7-15 days. Vitamin D3 100,000 IU on day 1 to augment the effects of resveratrol.<br><br>Placebo Group: Placebo tablets will contain brown rice flour in visually identical capsules, plus Vitamin D3 100,000 IU on day 1.	Phase 2; Terminated due to feasibility
NCT04403646	Tannin Specific Natural Extract for COVID-19 Infection	Double-blind, randomized, triple-masked, interventional clinical trial with parallel assignment	Tannins have been shown to have antioxidant, anti-inflammatory, antimicrobial properties and a regulatory effect on the gastrointestinal metabolism.	To assess the effect of treatment with a dietary supplement (ARBOX), a molecular complex of quebracho and chestnut tannins extract and Vit B12	124 COVID-19 positive participants ≥ 18 yrs.	Tannins	14 days	Two 390 mg ARBOX (dry extract of polyphenols (tannins) form quebracho and chestnut 240 mg, B12 vitamin 0.72 µg) capsules per day for 14 days plus conventional therapy.<br><br>Placebo Group: Placebo plus conventional therapy.	n/a; Terminated due to low number of COVID-19 patients
NCT04410510	P2Et Extract in the Symptomatic Treatment of Subjects With COVID-19	Double-blind, randomized, triple-masked, interventional clinical trial with parallel assignment	Antioxidants such as polyphenols show therapeutic effects in respiratory pathologies, leading to a decrease in clinical severity and suppression of inflammation, the latter may be related to the inhibition of NF-kB by polyphenols**.**<br><br>The standardized P2Et extract obtained from *C. spinosa*, is	To evaluate the efficacy of P2Et in reducing the length of hospital stay of patients with clinical suspicion or confirmed case of COVID-19.	100 COVID-19 positive participants ≥ 18 yrs.	P2Et (*Caesalpinia spinosa* extract)	14 days	P2Et active extract capsule equivalent to 250mg of P2Et every 12 hours for 14 days, plus standard care<br><br>Control: placebo capsule equivalent to intervention, plus standard care	Phase 2/3

			highly antioxidant and is said to decrease lipid peroxidation and tissue damage and induce complete autophagy in stressed or tumor cells. The induction of a full autophagic flow may inhibit the replication of beta-coronaviruses like SARS-CoV-2.						
NCT04844658	Covid-19, Hospitalized, Patients, Nasafytol	Standard-of-care comparative, open-labelled, parallel two-arms and randomized trial	NASAFYTOL®: Dietary supplement contains a highly bioavailable mixture of turmeric extract or curcumin (hydrophobic polyphenol of rhizomes of *Curcuma longa* L.), natural quercetin from *Sophora japonica* L. and vitamin D3 (at 60 % of the nutrient reference values); it is based on Polysorbate 80 and is designed to increase the bioavailability of the active components in humans. To improve the stability of the active ingredients in the capsule, the presence of a weak acid (citric acid) is essential.	To evaluate the efficacy and safety of NASAFYTOL® on COVID-19 positive hospitalized patients as a supportive treatment to standard-of-care in improving clinical parameters safely during hospital admission	50 COVID-19 positive participants ≥ 18 yrs.	Quercetin and Curcumin	Up to 14 days depending on duration of symptoms.	8 capsules of NASAFYTOL® taken orally per day (4 capsules in the morning and 4 capsules in the evening) in support of the standard COVID-19 treatment.<br><br>Control: 1 capsule of Vitamin D as FULTIUM® -D3 800 taken orally per day (1 capsule in the morning) as a supportive treatment to standard treatment	Recruiting
NCT04861298	Study to Investigate the Clinical Benefits of Dietary Supplement Quercetin for Managing Early COVID-19 Symptoms at Home	Open-labelled, randomized, parallel-assignment, interventional trial	Quercetin is established to have antioxidant and anti-inflammatory properties, which enable its antiviral, anticancer and anti-allergic activities; it also helps fight hay fever, arthritis, respiratory diseases, atherosclerosis and other cardiovascular and metabolic disorders. The flavonoid is also reported to stop radicals and reduce inflammatory gene expression. In addition, specific antiviral activity of quercetin, including against SARS-CoV-1, is also reported.	To explore the potential benefits of quercetin towards prevention of COVID-19 disease progression and improving symptoms in the early stage of infection.	142 COVID-19 positive participants ≥ 18 yrs.	Quercetin	14 days	Quercetin Phytosome (QP): 600 mg Quercetin daily for 1st week and 400 mg for the 2nd week, plus standard care<br><br>Control: standard of care	Recruiting

NCT04487964	Complementary Intervention for COVID-19	Open-labelled, non-randomized, parallel assignment, pilot interventional study	TCMs were recommended during the early stages of the COVID-19 pandemic in China given their effectiveness in prior outbreaks. Boswellia Serrata gum and Licorice extract, rich in flavonoids among other components, are reported to have antiviral, anti-inflammatory, anti-lung injury, antibacterial activity, antithrombic formation, and immunomodulatory activities; these properties may be helpful as medical intervention for COVID-19.	To investigate the therapeutic role of Licorice extract and Boswellia serrate gum as a complementary intervention in addition to conventional therapy in Egyptian patients with COVID-19	70 COVID-19 positive participants ≥ 18 yrs.	Largely liquiritin, isoliquiritin, liquiritin apioside, and licuraside	10-15 days	Licorice capsules: 250 mg standardized extract (25% Glycyrrhizin - 62.5 mg) twice daily for 10 days and *Boswellia serrata* 2gm Gum Resin: Volatile oils (5-15%), pure resin (55-66%), and mucus (12-23% 4 times daily for 15 days	Recruiting
NCT04468139	The Study of Quadruple Therapy Zinc, Quercetin, Bromelain and Vitamin C on the Clinical Outcomes of Patients Infected With COVID-19	Open-labelled, single-assignment, interventional trial	Higher intracellular levels of Zinc, as a mineral element that regulates adaptive immune cell function, is reported to decrease RNA-viral replication by affecting RdRp. Similarly, Quercetin, found in various natural sources, has anti-oxidant and anti-inflammatory properties; it is also reported to chelate zinc ions and act as zinc ionophore to cause zinc influx intracellularly. Bromelain has an immunomodulatory effect and decreases the majority of inflammatory mediators. Further, vitamin C is both an important co-factor physiologically, but is also an anti-oxidant, whose deficiency may lead to several organ injury.	To investigate the inhibitory effect of these molecules against viral replication, severity of the disease, cytokine storm and thrombosis.	60 COVID-19 positive participants ≥ 18 yrs.	Quercetin, bromelain		500 mg quercetin, 500 mg bromelain, 50 mg Zinc and 1000 mg Vitamin C daily doses for 5-10 days or until discharge	Phase 4
NCT04680819	Evaluation of the Effect of Anatolian Propolis on Covid-19 in Healthcare Professionals	Observational, prospective cohort study	No internationally consensual post-exposure prophylaxis for COVID-19 high-risk individuals exist currently. Propolis is a plant-derived natural resin	To investigate whether administration of Anatolian *propolis* can	50 HCWs at risk for developing COVID-19	3-O-Methylquercetin		20 drops of *Propolis* drop form twice daily for 1 month.	Not yet recruiting

	produced by bees and is reported to have anti-inflammatory, immunomodulatory and anti-oxidant properties. Multiple stages of the SARS-CoV-2 infection mechanism are thus potential targets. Spectrophotometry reports the existence of 73.1 mg flavonoids per ml extract.	prevent the development of COVID-19 in people at risk, particularly HCWs, thereby reducing the morbidity and mortality of the outbreak.		Control: no such preventive measures.

NLR: Neutrophil to Lymphocyte Ratio; hs-CRP: high-sensitivity C-reactive protein; ARDS: acute respiratory distress syndrome; PDI: Protein disulfide-isomerase.
